# Lichens—A Potential Source for Nanoparticles Fabrication: A Review on Nanoparticles Biosynthesis and Their Prospective Applications

**DOI:** 10.3390/jof7040291

**Published:** 2021-04-12

**Authors:** Reham Samir Hamida, Mohamed Abdelaal Ali, Nabila Elsayed Abdelmeguid, Mayasar Ibrahim Al-Zaban, Lina Baz, Mashael Mohammed Bin-Meferij

**Affiliations:** 1 Molecular Biology Unit, Department of Zoology, Faculty of Science, Alexandria University, Alexandria 21500, Egypt; reham.hussein@alexu.edu.eg (R.S.H.); dr_nabila_elsayed2000@yahoo.com (N.E.A.); 2 Biotechnology Unit, Department of Plant Production, College of Food and Agriculture Science, King Saud University, Riyadh 11543, Saudi Arabia; mali3@ksu.edu.sa; 3 Plant Production Department, Arid Lands Cultivation Research Institute, City of Scientific Research and Technological Applications (SRTA-City), New Borg El-Arab, Alexandria 21934, Egypt; 4 Department of Biology, College of Science, Princess Nourah bint Abdulrahman University, Riyadh 11543, Saudi Arabia; mmbinmufayrij@pnu.edu.sa; 5 Department of Biochemistry, Faculty of Science, King Abdulaziz University, Jeddah 21589, Saudi Arabia

**Keywords:** lichen, nanoparticles, green synthesis, eco-friendly, antimicrobial, antioxidant

## Abstract

Green synthesis of nanoparticles (NPs) is a safe, eco-friendly, and relatively inexpensive alternative to conventional routes of NPs production. These methods require natural resources such as cyanobacteria, algae, plants, fungi, lichens, and naturally extracted biomolecules such as pigments, vitamins, polysaccharides, proteins, and enzymes to reduce bulk materials (the target metal salts) into a nanoscale product. Synthesis of nanomaterials (NMs) using lichen extracts is a promising eco-friendly, simple, low-cost biological synthesis process. Lichens are groups of organisms including multiple types of fungi and algae that live in symbiosis. Until now, the fabrication of NPs using lichens has remained largely unexplored, although the role of lichens as natural factories for synthesizing NPs has been reported. Lichens have a potential reducible activity to fabricate different types of NMs, including metal and metal oxide NPs and bimetallic alloys and nanocomposites. These NPs exhibit promising catalytic and antidiabetic, antioxidant, and antimicrobial activities. To the best of our knowledge, this review provides, for the first time, an overview of the main published studies concerning the use of lichen for nanofabrication and the applications of these NMs in different sectors. Moreover, the possible mechanisms of biosynthesis are discussed, together with the various optimization factors influencing the biological synthesis and toxicity of NPs.

## 1. Introduction

Nanotechnology has recently created a revolution in the scientific world, particularly in the industrial, medical, agricultural, and electronic sectors [1,2]. This revolution is due to the ability of this technology to generate new products in the nanoscale (at least one of their dimensions in the range 1–100 nm) with unique and desirable physicochemical and biological characteristics that are missing in their precursor forms [3,4]. These nanomaterials (NMs) have a high surface-area-to-volume ratio and can therefore be used in drug delivery [5], catalysis [6], therapeutics [7,8], theranostics [9], and detection and diagnostic fields [10]. For instance, the specific surface properties, porosity, and ability for functionalization render silica nanoparticles (NPs) appealing options for drug delivery [11]. Fullerenes can be loaded with different therapeutic agents such as antibiotics and anticancer drugs [12,13]. Silver NPs show unique reactivity, selectivity, and stability, as well as recyclability in catalytic reactions [14]. Magnetic NPs have a high magnetic moment and consequently are attractive tools for magnetic resonance imaging for cancer diagnostic [15].

Furthermore, the smaller size of NPs has facilitated the development of new therapeutic agents against serious global illnesses such as cancer and infectious and parasitic diseases [11]. Platinum [16], selenium [17], and palladium NPs acted as potent anticancer agents [18], while zinc oxide [19], copper oxide [20], and titanium dioxide NPs exhibited significant inhibitory activity against different microbes including bacteria, fungi, and viruses [21,22]. NPs also play important roles in communication and electronic fields due to high electro-optical activity, enabling them to be used in electronic and optical industries [23,24]. The thermal conductivity of NPs provides scope for researchers to develop numerous energy cells such as solar cells and batteries [25,26], while the unique photothermal properties of NPs mean they are a promising therapeutic for various types of cancers [27]. The localized surface plasmon resonance (SPR) of gold NPs enable these particles to absorb specific wavelengths, leading to photoacoustic and photothermal characteristics; consequently, these NPs are promising tools for hyperthermic cancer therapies and bioimaging [27].

These unique features of NPs facilitate the creation and development of new tools, processes, and products with applications in many sectors, including medicine, industry, and communication; thus, enthusiasm for producing novel nanoproducts has rapidly increased. However, this swift growth has resulted in harmful effects on living organisms and their environments [4,28]. This damage arises from using and yielding hazardous materials during the production of NPs via chemical synthesis approaches. Moreover, the approach of using physical methods in the fabrication of NPs consumes more energy and money than other synthesis methods. To minimize these drawbacks, eco-friendly alternatives to traditional synthesis methods (chemical and physical routes) have been sought. One of these alternatives is the green fabrication of NPs.

Biofabrication, biological synthesis, green synthesis, and biosynthesis are synonymous terms corresponding to the use of eco-friendly, rapid, simple, and low-cost technology for NP production. This technology has numerous advantages, including high scalability, variation in size/shape and chemical compositions, and high mono-dispersity of NPs [29]. Moreover, this approach uses living organisms or their products to reduce bulk materials into NPs and stabilize the NPs without needing chemical materials or producing any hazardous materials [4]. Multicellular and unicellular organisms (plants, algae, worms, lichens, fungi, bacteria, cyanobacteria, yeast, actinomycetes, etc.) and their biomolecules, such as proteins, pigments, enzymes, vitamins, polysaccharides, and lignin were used as reductants and surfactants for fabricating precursors into their nanoforms [4,30,31,32]. These biogenic NPs can be consumed in numerous industrial and medical processes due to their unique physicochemical and biological features such as efficiency, biocompatibility, bioactivity, and stability [33].

Although there are limited reports about the lichen-based green synthesis of NPs, this method is considered a promising technology for NP production. Lichens are composite organisms, which live in both obligate and beneficial symbiosis with fungi, algae, perennial trees, or cyanobacteria [34]. Lichen cells contain many types of secondary metabolites and other bioactive molecules, rendering them valuable for industrial, pharmaceutical, biotechnological, medical, and cosmetics applications [35]. Some researchers have demonstrated the potentiality of different species of lichens to fabricate unique NPs with different shapes, sizes, and physicochemical and biological activities [‎^36^]. Rattan et al. demonstrated the role of different lichen species to synthe-size different types of NMs and their potentiality to act as promising antimicrobial agents [‎^36^]. Alqahtani et al. reported that methanolic extracts of two lichen species, *Xanthoria parietina* and *Flavopunctelia flaventior*, were recently shown to have the po-tential to reduce silver nitrate into Ag-NPs extracellularly [‎^37^]. The resultant Ag-NPs were spherical, had a nanosize range of 1–40 nm, and reduced the proliferation of human colorectal cancer (HCT 116), breast cancer (MDA-MB-231), and pharynx can-cer (FaDu) cell lines, and the growth of methicillin-resistant Staphylococcus aureus (MRSA), vancomycin-resistant Enterococcus (VRE), Pseudomonas aeruginosa, and Esche-richia coli [‎^37^]. This review provides, for the first time, an overview of the main published studies concerning the use of lichen for nanofabrication and the applications of these nanomaterials in different sectors. Moreover, the possible mechanisms of biosynthesis are discussed together with the various optimization factors influencing the biological synthesis and toxicity of NPs.

## 2. Classification of Nanoparticles

**Figure 1:**
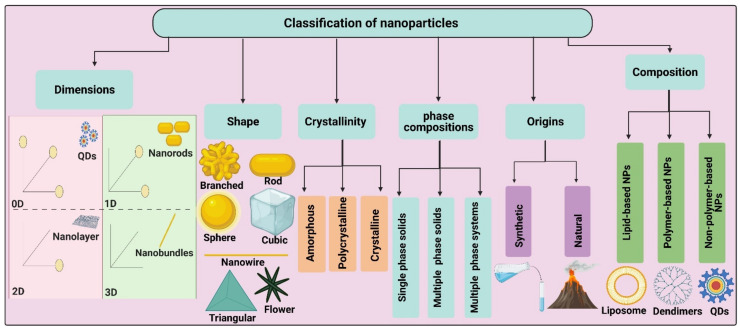
Classifications of nanoparticles (NPs).

Classification of NPs varies according to their origins, structures, shapes, dimensions, chemical and phase compositions, physical and chemical properties, and crystallinity [29,38,39]. For example, NPs can be obtained naturally from dust, volcanic eruptions, and living organisms such as bacteria, fungi, algae, plants, etc., and artificially by utilizing chemical, physical, and biological synthesis routes such as colloidal, chemical precipitation, laser ablation, sputtering, and micro- and macro-organism-mediated synthesis approaches [30,31,40]. Furthermore, NPs can be classified into organic, inorganic, and semi-organic types according to their chemical nature [29,41]. Similarly, NPs can be categorized according to their shape, including rod, spherical, cubic, triangular, octahedral, pentahedral, flower, star, etc. [4,39], and on the basis of the number of their dimension in nanoscale, including zero dimension (0D) such as quantum dots, one dimension (1D) such as nanowires and nanorods, two dimensions (2D) such as nanolayers and nanoplates, and three dimensions (3D) such as nanocoils and nanoflowers [42]. In terms of magnetic properties, NPs can belong to either the paramagnetic category, which includes iron oxide and zinc sulfide NPs, or the diamagnetic category comprising titanium oxide and magnesium ferrite NPs [43] (Figure 1).

## 3. Synthesis Routes of Nanoparticles

**Figure 2:**
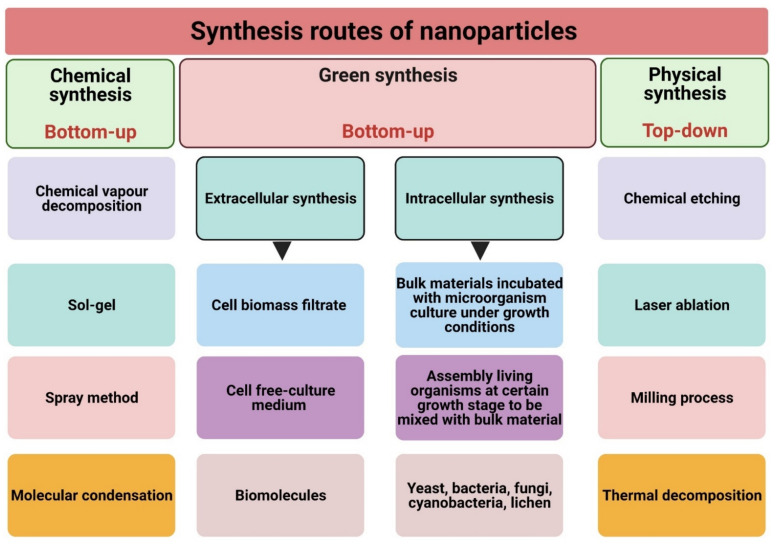
Synthesis routes of nanoparticles.

Nanofabrication routes can generally be classified into two main groups: top-down methods, such as physical synthesis approaches, and bottom-up synthesis methods, such as chemical and biological synthesis processes (Figure 2) [44].

### 3.1. Physical Synthesis

This route is a top-down synthesis process in which precursors are reduced into NPs using physical approaches such as ultra-sonication, laser ablation, mechanical milling, sputtering, microwave irradiation, and electrochemical methods [31]. Quantum dot NPs have been physically synthesized using molecular beam epitaxy, ion implantation, e-beam lithography, and X-ray lithography [45,46]. Recently, carbon nanostructures were prepared from elemental graphite powders using a mechanical milling method in air [47]. Niasari et al. synthesized silica NPs using rice husk ash at ambient temperature by utilizing a high-energy planetary ball mill [48]. The scholar reported that silica NPs were synthesized after 6 h of ball milling. Fe-SEM and transmission electron microscopy (TEM) micrographs exhibited that silica NPs have a spherical shape and nanosize of 70 nm. They reported that silica NPs acted as a promising drug delivery system for controlling penicillin-G drug releasing. Recently, simple green- microwave-assisted synthesis route of fluorescent carbon quantum dots (CQDs) was conducted using roasted chickpea as the carbon source in one step without using any chemicals [49]. The study provided an eco-friendly method to fabricate CQDs with advantageous properties such as high fluorescence intensity, excellent photostability, and good water solubility. The physicochemical features of CQDs were determined using UV–Vis spectroscopy, fluorescence spectroscopy, Fourier transform infrared spectroscopy (FTIR), X-ray diffraction (XRD), transmission electron microscopy (TEM), and selected-area electron diffraction based on TEM micrographs. The data revealed that CQDs emitted blue fluorescent at a UV wavelength of 365 nm and have a spherical shape with an amorphous structure and nanodiameter less than 10 nm.

### 3.2. Chemical Synthesis

Chemical synthesis is a bottom-up process in which atoms are assembled into nuclei and grown to NPs [50]. The predominant components of this route are the reducing (such as sodium citrate and ascorbate) and capping agents (such as sodium carboxyl methylcellulose) [51,52]. Chemical vapor deposition, spinning, pyrolysis, and sol-gel process are examples of chemical synthesis approaches [41]. Titanium (Ti) dioxide NPs were fabricated from the precursor Ti-isopropoxide and calcined at 300, 350, 400, and 450 °C using a simple sol-gel method [53]. The chemical reduction was used to reduce silver nitrate into Ag-NPs using sodium citrate (TSC) and sodium borohydride (NaBH_4_) as reducing agents [54]. The authors used the face-centered central composite model with four abiotic parameters including AgNO_3_, TSC, and NaBH_4_ concentrations and the pH of the reaction. They revealed that optimal conditions to synthesize spherical Ag-NPs with a nanosize of less than 10.3 nm were pH 8 and 0.01 M, 0.06 M, 0.01 M for the concentration of TSC, AgNO_3_, and NaBH_4_, respectively. Yu et al. synthesized hollow silica spheres (HSSs) through a self-templating route in acidic aqueous media under hydrothermal conditions [55]. The resultant HSSs have a spherical shape with a nanodiameter of 190 nm. They found that the hollowed-out interior space of HSSs was dependent on reaction time and silica concentration, while their porous structure in the shell can be mitigated by tuning the acidity of the silica dispersion. Moraes et al. synthesized tadpole-like gold nanowires (AuNWs) by mixing 0.1 mmol of HAuCl_4_·3H_2_O with 12 mL of the oleylamine as a reducing agent at 65 °C under stirring for 72 h [56]. The scholar exhibited that AuNWs were polydispersed and branched with length ranging from a few nanometers to larger than 500 nm with a diameter of 23 nm.

### 3.3. Biological (Green) Synthesis

Biological synthesis is a modern alternative to both physical and chemical synthesis processes and is considered a type of bottom-up route [57]. This approach utilizes natural sources such as microorganisms, macroorganisms, and biomolecules (proteins, lipids, polysaccharides, pigments, etc.) to fabricate NPs from their bulk materials without the need for toxic chemicals during the fabrication process [4,58]. Various significant properties of biosynthesis routes such as the absence of poisonous chemical compounds used as reducing or stabilizing agents, no toxic yields generated from the process, the low energy consumption, inexpensive cost, and high scalability have resulted in green synthesis methods becoming more attractive than other traditional methods [4].

Biological synthesis routes are categorized into two main approaches—extracellular and intracellular synthesis routes.

#### 3.3.1. Extracellular Synthesis

**Figure 3:**
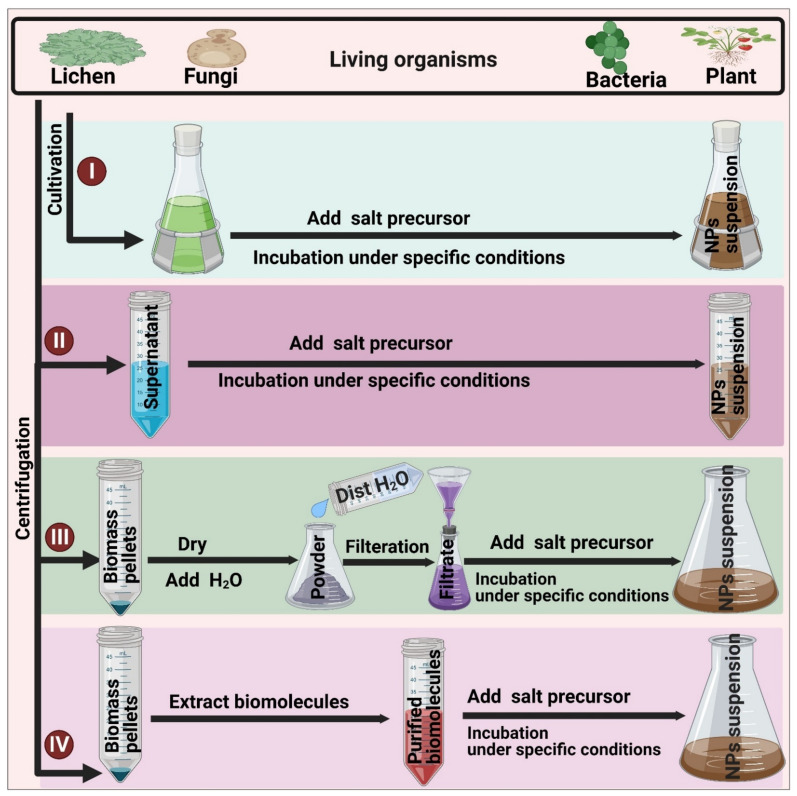
Green synthesis methods include intracellular synthesis route (**I**) and extracellular synthesis routes including cell-free, culture-medium-based synthesis of NPs (**II**), cell–biomass-filtrate synthesis of NPs (**III**), and biomolecule-mediated synthesis of NPs (**IV**).

In extracellular synthesis routes, the fabrication process occurs outside living cells [57,59]. This process can be achieved via three different patterns:

(i) Cell-biomass-filtrate synthesis of NPs: In this pattern, cells of living organisms are dried with a lyophilizer, oven, or air-based methods and then crushed into fine powders that are mixed with distilled water for boiling. The mixture is cooled, passed through a filtration system such as Whatman filter paper, and then the resulting filtrate is mixed with a defined concentration of bulk material to fabricate it into NPs [28,58,60]. An alternative procedure involves washing the natural sources such as *Streptomyces* sp., algae, etc., then soaking the cells in water for a number of days, followed by centrifugation and use of the resulting supernatant as a reducing and stabilizing agent to synthesize NPs [61,62]. Other methods achieved the extracellular synthesis of NPs by sonicating or boiling the natural sources under certain conditions, then filtering the mixture and using the filtrate in the synthesis process [33,63];

(ii) Cell-free, culture-medium-based synthesis of NPs: This method is suitable for cultured microorganisms. First, the culture is centrifuged and the supernatant used for bioreduction of bulk compounds into their NPs under suitable conditions. Keskin et al. demonstrated that cell-free culture media of *Synechococcus* sp. had a reducible activity that resulted, under light conditions, in the formation of Ag-NPs with an average nanosize of 140 nm [64]. However, this process is sometimes unsuitable for NP synthesis because many types of media used for the culture of microorganisms contain components that act as reductants and stabilizing agents. These compounds interfere with the reducible activity of active biomolecules of the cultured microorganisms [65];

(iii) Biomolecule-mediated synthesis of NPs: This approach uses biomolecules such as pigments, carbohydrates, proteins, enzymes, etc. as reducing and capping materials to produce NPs. Briefly, target biomolecules are extracted from their micro- or macro-organisms, purified, and mixed with a defined concentration of bulk material solutions to start the NP fabrication process under specific conditions of temperature, illumination, and pH [66,67] (Figure 3).

#### 3.3.2. Intracellular Synthesis

The intracellular synthesis method refers to the production of NPs inside living cells, with biological processes such as metabolic activity, respiration, and growth stage, playing crucial roles in the biosynthesis process [68]. Intracellular synthesis can be performed according to two protocols, each composed of three steps: (i) culturing the target living organism, (ii) the reaction between precursor materials and living cells, and (iii) separation and purification of NPs and subsequent characterization using different physicochemical methods [4,69]. The first protocol includes the incubation of bulk materials solution with microbe cultures during their growing period under standard culture conditions until the microbes reach a certain growth [70]. In the second method, living cells in the logarithmic phase are collected by centrifugation, washed multiple times to discard any undesired materials, and then the cleaned microbial biomass is dissolved in water and mixed with a suitable amount of bulk material solution [71].

Intracellular synthesis is more complicated than extracellular fabrication due to the additional steps required to extract and purify NPs from inside the cells [72]. Both types of biological synthesis methods are eco-friendly routes, do not usually need toxic chemical materials, and are easily performed under normal laboratory conditions [4,65] (Figure 3).

## 4. Green Synthesis-Based Systems

### 4.1. Biomolecule-Mediated Fabrication of NPs

Recently, natural products have become a target area for many researchers due to their promising applications in numerous sectors including biotechnology (e.g., biofuel and biofertilizer production), bioremediation, and the cosmetics industry (e.g., synthesis of natural sunblock creams). Moreover, natural biomolecules exert broad biomedicinal and therapeutic potentials for serious diseases including cancers and infectious, parasitic, and immune diseases, etc. [7,73]. For instance, scytonemin, a natural pigment extracted from cyanobacteria *Scytonema* sp., is an extremely potent modulator of mitotic spindle formation [74]. In addition, calothrixins, quinone-based natural products extracted from cyanobacteria *Calothrix* sp., exhibit potent antiproliferative activity against cancer cell lines [75]. Normavacurine-21-one, isolated from *Alstonia scholaris* leaves, displays antibacterial activities against *Enterococcus faecalis* ATCC 10541. Conversely, biomolecules exhibit significant reducible properties and thus have the ability to fabricate numerous metal precursors into their nanoforms [76].

Biomolecules such as proteins, amino acids, or secondary metabolites from microorganisms or plant extracts, can act as reduction, stabilization, functionalization, and capping agents for NPs [77]. For the green synthesis of metal NPs, aqueous extracts of dried plants or algae are commonly used [33,78]. The water extract classically contains phenolics, terpenoids, polysaccharides, flavonoids, alkaloids, lipids, proteins, and carbohydrates, which collectively represent the reducing power needed for the process. Generally, plant extracts contain enzymes and amino acids that can act as reductants for silver ions and are therefore utilized as scaffolding to facilitate the formation of silver NPs [79]. This unique synthesis strategy provides several kinds of functional groups for NP functionalization [80]. Numerous studies have attributed the synthesis mechanisms for NPs to the potentiality of biomolecules to reduce and stabilize NPs, thereby providing more provision to improve and control the shape, size, and crystallinity of nanomaterials [81].

#### 4.1.1. Pigments

A vital constituent of most photosynthetic organisms is the pigments, including chlorophylls, carotenes, and anthocyanins [82]. Natural pigments produced by plants, algae, and microorganisms are distinctive biomolecules that have been used in the biological synthesis of NPs. Although studies on the use of biopigments for bioreduction of NPs are limited, these biopigments are known to act as potent reducing and stabilizing agents during biofabrication of NPs [80]. Photosynthetic accessory pigments, such as carotenoid, cochineal, flexirubin, fucoxanthin, melanin, phycocyanin, and C-phycoerythrin and R-phycoerythrin, are the predominant pigments in many organisms, including cyanobacteria, microalgae, actinomycete, algae, etc., and have been extensively exploited in the synthesis of NPs [4,66,83].

Actinorhodin isolated from *Streptomyces coelicolor* successfully reduced silver nitrate (AgNO_3_) into stable Ag-NPs [84]. El-Naggar et al. synthesized Ag-NPs using phycocyanin extracted from *Nostoc linckia* and studied the anticancer, antibacterial, and antihemolytic activities of these NPs [66]. The blue pigment was observed to be an efficient reductant and surfactant material for the production of Ag-NPs. Moreover, pigment-coated Ag-NPs exhibited significant antitumor properties against MCF-7 cell lines, with an IC_50_ of 27.79 ± 2.3 µg/mL, and act as a tumor progression suppressor against Ehrlich ascites carcinoma-bearing mice. Green pigment extracted from Alfalfa plant leaves extracellularly reduced AgNO_3_ into Ag-NPs [82]. The particle size of the resultant quasi-spherical biogenic Ag-NPs was 25 nm, and the reducible activity of the green pigments was attributed to chlorophylls and carotenes.

The pigment produced by *Talaromyces purpurogenus* was also used as a reducing agent to manufacture Ag-NPs [85]. A reaction mixture (5 mL) was prepared by mixing 0.5 g/L of extracted pigment with 2 mM AgNO_3_ and adjusting the pH to 12 using 5 N sodium hydroxide solution. The mixture was vortexed then incubated at 28 °C with 2000 lux of light for 48 h. The formation of Ag-NPs was monitored by color change from light orange to brown and by UV–visible (UV–Vis) spectroscopy detection. The UV–Vis spectrum displayed a peak at 410 nm, the known SPR of Ag-NPs. The size of the resulting NPs was in the range of 4–41 nm. To investigate the functional groups present in the pigment, Fourier transform infrared spectroscopy (FTIR) analysis was conducted at a fixed pH of 12, the conditions in which Ag-NPs were generated. At alkaline pH, phenolic groups were reported to donate electrons that reduce the silver ions to Ag-NPs.

#### 4.1.2. Carbohydrates

Polysaccharide-based green synthesis of NPs has been a more attractive method in nanobiotechnology due to the stability, hydrophilicity, nontoxicity, bioactivity, and biodegradable properties of these NPs [86]. Ebrahiminezhad et al. synthesized Ag-NPs using the carbohydrate secreted by *Chlorella vulgaris* [87]. The resulting green Ag-NPs were uniformly dispersed and spherical shaped, with an average size of 7 nm and positive zeta potential of +26 mV. The authors suggested the carbohydrate coat surrounding the Ag-NPs was 2 nm based on a comparison between the size of the NPs in transmission electron microscopy (TEM) micrographs (7 nm) and their hydrodynamic diameter (9 nm).

Palladium NPs (Pd-NPs) have been fabricated from palladium chloride using carboxymethyl cellulose as a reducing and capping agent at 80 °C for 30 min [88]. The Pd-NPs were spherical with a crystallinity structure and an average size of 2.5 nm. Pd-NPs have a negative zeta potential value of −52.6 mV, which is indicative of their high stability. Furthermore, the biogenic Pd-NPs showed high catalytic activity against azo-dyes.

#### 4.1.3. Enzymes

Enzymes are complex globular proteins present in living cells where they act as catalysts to facilitate chemical changes in substances. With the development of biochemistry came a fuller understanding of the wide range of enzymes present in living cells and their modes of action [89]. Although enzymes are only formed in living cells, many can be extracted or separated from the cells and can continue to function in vitro. This unique ability of enzymes to perform their specific chemical transformations in isolation has led to the use of enzymes in industrial and food processes, bioremediation, and medicine [90]. Furthermore, enzymes are nontoxic and biodegradable, making them environmentally friendly and attractive for medical applications [91]. All these characteristics of enzymes, plus their unique and precise structure, have rendered them desirable for green synthesis of NPs [31]. A prime example is the synthesis of Au-NPs by the action of extracellular amylase from *Bacillus licheniformis* on AuCl_4_ at pH 8 [92]. Another example is the sulfite reductase enzyme extracted from *E. coli* by ion-exchange chromatography and used for the production of Au-NPs that exhibit antifungal activity [93]. NADH and NADH-dependent enzymes were investigated for their role in the biosynthesis of metal NPs. These extracellular enzymes are highly effective reducing agents due to their ability to shuttle electrons in the reduction process of metals to produce NPs [94,95].

#### 4.1.4. Proteins

NP biosynthesis in the presence of proteins from several biological sources can produce NPs with uniform size and shape and minimal particle aggregation. In these processes, the functional groups of proteins act as the reducing and capping agents to metal ions [96,97]. Proteins were utilized in the bioreduction, capping, and assembly of selenium oxyanion, contributing to controlling the size and morphology of selenium NPs (Se-NPs) [98]. Proteins are crucial in the reduction of selenites and selenates and the stabilization of Se-NPs, which exhibit a unique nanostructure contrary to those obtained chemically [98]. Sanghi et al. found that the production of Au-NPs was facilitated by proteins of the fungus *Coriolus versicolor* [99]. Characterization of these Au-NPs by UV–Vis spectroscopy, scanning electron microscopy (SEM), and atomic force microscopy (AFM), revealed that the NPs had high stability (they can be stored up to six months without any aggregation) and a size of 5–30 nm. FTIR data demonstrated the crucial role of different fungal proteins in the fabrication of Au-NPs. A study in 2018 reported the synthesis of Au-NPs with high stability by using the supernatant of fermented fungi containing the extracellular proteins [65]. This process resulted in the formation of Au-NPs with sizes ranging from 6 to 40 nm.

#### 4.1.5. Lipids

Mannosylerythritol lipids were used as a reducing and stabilizing agent in the green synthesis of Ag-NPs [100]. The process commenced with the addition of 0.01 g mannosylerythritol lipids to 1 mL acetone diluted with 10 mL dechlorinated water; pH of the whole solution was adjusted to 7 utilizing 0.1 M sodium hydroxide. The solution was added dropwise to 100 mL of 2 mM silver nitrate solution and kept at room temperature with continuous stirring. The mixture changed from pale-yellow to brownish-red, and the UV–Vis absorption spectrum of the synthesized Ag-NPs was recorded at 430 nm. This confirmed that mannosylerythritol lipids were effective as reducer and stabilizer agents in the formulation of Ag-NPs. An energy dispersive spectroscopy (EDS) instrument equipped with the SEM was used to determine the chemical composition, size, and morphology of Ag-NPs. The structure of the Ag-NPs was perceived by TEM after dispersing powdered NPs in methanol and sonicating the solution. The TEM structure provided more information about the crystallinity and average size of the Ag-NPs.

X-ray diffraction (XRD) of the produced Ag-NPs showed four characteristic peaks of 28.4°, 33.2°, 47.4°, and 56.3° at 2θ, which correspond to the lattice planes (111), (200), (220), and (311), respectively, confirming the crystalline and face-centered cubic (fcc) structure of the NPs. Meanwhile, the FTIR spectrum of the mannosylerythritol lipids Ag-NPs demonstrated significant peaks at 3337, 2923, 1742, 1562, 1344, 1093, 718, and 534 cm^−1^, which indicate the presence of various functional groups in the mannosylerythritol glycolipid capping the Ag-NPs. The peak at 3337 cm^−1^ may be due to –OH from polysaccharides, while the peak at 2923 cm^−1^ might indicate (C–H) stretching of alkanes. The strong band at 1562 cm^−1^ could be due to the carbonyl stretching vibration. The peaks at 1466 and 1344 cm^−1^ can be assigned to (C–N) and (C–C) stretching vibration of aromatic and aliphatic amines, while the band at 1093 cm^−1^ could be assigned to (C–O) of alkoxy groups, and peaks at 718 and 534 cm^−1^ to CH_2_ groups.

A different study used *Lactobacillus casei* to synthesize of Au-NPs and the *L. casei* components were compared before and after the addition of auric acid (0.5 mM K[AuCl_4_]) [101]. The levels of unsaturated lipids decreased significantly after the addition of auric acid. Moreover, the formation of Au-NPs caused a reduction in the levels of diglycosyldiacylglycerol (DGDG) and triglycosyldiacylglycerol (TGDG). DGDG extracted from *L. casei* induced the formation of Au-NPs, suggesting that these glycolipids can act as potent reducing agents for the conversion of Au(III) to Au(0) and that results in the formation of small NPs.

#### 4.1.6. Vitamins

The utilization of vitamin B2 as a reducing and capping agent in the green synthesis of Ag and Pd nanowires and nanorods is a distinctive technique in the field of green nanotechnology [102]. Ascorbic acid (vitamin C) is used as a reducing factor in combination with chitosan as a stabilizing agent to fabricate sodium alginate-silver NPs [103]. Malassis et al. demonstrated a prompt and effective method to fabricate Au-NPs and Ag-NPs by exploiting ascorbic acid as a reducing and stabilizing agent [104]. The size of the NPs produced was 8–80 nm for Au-NPs and 20–175 nm for Ag-NPs. The method yielded versatile NP surface modification with a large variety of water-soluble surfactants that can be neutral, positively, or negatively charged. Ahmed et al. reported that ascorbic acid in *Desmodium triflorum* was the predominant biomolecule in the reduction process for Ag-NPs [105].

Production of Se-NPs coated with ascorbic acid was achieved through the bioreduction of selenite (Na_2_SeO_3_) [106]. Selenite was mixed with ascorbic acid and the mixture turned orange red after 30 min, confirming the fabrication of Se-NPs. The produced Se-NPs were analyzed by TEM and dynamic light scattering (DLS) and were observed to have an average size of 23 ± 5.0 nm. These NPs were shown to be an excellent candidate for radiopharmaceutical imaging techniques used in the diagnosis of liver and kidney cancers.

Another important vitamin exploited for the synthesis of NPs is vitamin B12. To synthesize Ag-NPs, Au-NPs, and Pd-NPs, vitamin B12 solution was mixed with silver nitrate, gold (III) chloride, and palladium acetate solutions, respectively [107]. All mixtures were tested in the presence and absence of microwave (MV) irradiation. The results exhibited that in the absence of MV irradiation, vitamin B12 did not reduce bulk material to their nanoform. However, MV irradiation enhanced the reduction ability of vitamin B12 to fabricate metals into NPs. XRD analysis of the resultant metallic NPs confirmed the efficiency of this vitamin as a reducing agent. The morphological features of the synthesized Ag-NPs, Au-NPs, and Pd-NPs were examined by using SEM and TEM techniques, and large aggregates with irregular shapes and diameters in the range 70–600 nm were observed. Ag samples treated with MW irradiation for 6 min produced NPs with diameters less than 30 nm. While Au samples treated with MV irradiation for 3 min showed irregular shapes and small-size particles with an average diameter of 40 ± 11.7; larger Au NPs with a diameter > 500 nm were observed after a longer period of irradiation (i.e., 6 min). Pd samples irradiated with MV for 3 min resulted in NPs with an average size of 40.2 ± 7.3 nm, whereas that irradiated with MV for 6 min produced two different diameters of 43.9 ± 7.1 and 6.6 ± 2.1 nm. The NPs were spherical, triangular, and decahedron shaped. It was concluded that MV irradiation duration is the key to mitigate noble NPs size.

#### 4.1.7. Secondary Metabolites

Secondary metabolites of different microorganisms, plants, and animal collagen waste were noted to have several properties that enhance the synthesis of NPs and could potentially be deployed in major pharmaceutical studies. Some of the notable secondary metabolites that serve as NP stabilizers include alkaloids, cardiac glycosides, flavonoids, phenols, tannins, and terpenoids [108,109,110]. Of these compounds, flavonoids are the most utilized secondary metabolites for green synthesis due to their practical structure and the favorable qualities they provide for human health. Pertaining to the flavonoid family are anthocyanins, which have been thoroughly investigated for their antioxidant activity [110]. One study tested the effects of anthocyanins as secondary metabolites on the green synthesis of Ag-NPs by using an aqueous extract of saffron wastage and reported a marked reduction of silver ions and antibacterial activity against several bacterial strains [109].

A study on Ag-NP synthesized using an aqueous extract of *Pteris tripartita* proved the anti-inflammatory activity of flavonoids-coated Ag-NPs by conducting an in vivo investigation on mice with edema, and reported a success rate of nearly 60% [111]. These findings provide an optimistic outlook for the future of NPs in biotechnology and drug discovery applications since they present an efficient way of producing metal NPs without chemical stabilizers or reducers through the use of abundant and natural compounds such as flavonoids, phenols, tannins, terpenoids, reducing sugars, and proteins.

### 4.2. Living Organisms-Mediated Fabrication of NPs 

Many micro- and macro-organisms are used as biofactories to produce NPs with unique physicochemical and biological activities.

#### 4.2.1. Plants

Plant-mediated fabrication of NPs, or phytonanotechnology, is a recognized branch of green synthesis of NPs due to being an eco-friendly, low-cost, rapid, and simple method. Other beneficial features of phytonanotechnology processes are their scalability, bioactivity, biocompatibility, and broad medical applicability [112]. Plant extracts act as reducing and capping agents for the synthesis of many types of NPs [33]. Different parts of plants, including leaves, fruits, stems, seeds, and roots, showed their reducing ability during the synthesis of metallic NPs [113,114]. Singh et al. successfully synthesized Au-NPs and Ag-NPs using *Panax ginseng* leaf and root extracts within 3 and 45 min at 80 °C [115]. Saratale et al. fabricated silver nitrate into Ag-NPs using *Acacia nilotica* leave extract as reducing and stabilizing agents to investigate their antineoplastic, free radical scavenging activity and sensing potency for H_2_O_2_ [116]. The scholars reported that Ag-NPs formed within 20 min of mixing 10 mL of plant leave extract to 100 mL of 1 mM AgNO_3_ solution. The resultant NPs have a spherical shape and nanosize range of 5 to 30 nm.

Krishnan et al. biosynthesized Ag-NPs from *Piper nigrum* extract and investigated their antitumor activity [117]. TEM images revealed that the Ag-NPs were spherical with a size of 20 nm. The cytotoxicity of Ag-NPs and *Piper nigrum* extract at various concentrations in the range of 10–100 μg/mL was investigated against breast and liver cancer cell lines (MCF-7 and HepG2 cells, respectively) and confirmed their potent cytotoxic effect. In a different study, biosurfactant extracted from corn steep liquor was used to biosynthesize Ag- and Au-NPs. The bioreduction process was completed in one step under a controlled temperature at 60 °C and resulted in a mixture of nanospheres and nanoplates. Biosurfactants were essential for the bioreduction process and also for stabilization of the produced NPs, which improved the antimicrobial activity of the NPs [118].

Green synthesis of Au-NPs by *Salicornia brachiata* (Sb) plant extract and characterization of the formed NPs revealed that mixing plant extract (50 mL) with 10 mM NaBH_4_ was sufficient to yield the purple color that indicated the formation of Sb-Au-NPs [119]. TEM micrographs showed that the size of Sb-Au-NPs was approximately 30 nm, while XRD and EDS data proved that Sb-Au-NPs had a pure crystalline form.

#### 4.2.2. Algae, Microalgae, Cyanobacteria, and Diatoms

Algae, microalgae, and cyanobacteria have emerged as attractive biofabrication machines for many NPs [4,68]. The synthesis and antimicrobial and antioxidant applications of Au- and Ag-NPs produced through the exploitation of cell-free extracts of the microalga *Neodesmus pupukensis* were explored [120]. Zone of inhibition tests showed that Ag-NPs were active against *Pseudomonas* sp. (43 mm), *Escherichia coli* (24.5 mm), *Klebsiella pneumoniae* (27 mm), and *Serratia marcescens* (39 mm). In contrast, Au-NPs only showed activity against *Pseudomonas* sp. (27.5 mm) and *Serratia marcescens* (28.5 mm). Antifungal tests indicated that Ag-NPs had mycelial inhibition of 80.6, 57.1, 79.4, 65.4, and 69.8% against *Aspergillus niger*, *A. fumigatus*, *A. flavus*, *Fusarium solani*, and *Candida albicans*, respectively, while Au-NPs had 79.4, 44.3, 75.4, 54.9, and 66.4% against *A. niger*, *A. fumigatus*, *A. flavus*, *F. solani*, and *C. albicans*, respectively. The free radical scavenging power of Au-NPs and Ag-NPs was 68.9 and 41.21%, respectively. The authors concluded that Au- and Ag-NPs fabricated by *Neodesmus pupukensis* have significant potential as antimicrobial and antioxidant agents and could be used for various biotechnological applications.

Colin et al. reported an eco-friendly green synthesis method to produce Au-NPs with enhanced biocompatibility [76]. The method used an extract from the alga *Egregia* sp., which naturally contains biomolecules that are important for shell formation around the Au-NPs to improve their biocompatibility. The algae extract functions as the reducing agent and as the stabilizing capping shell for the Au-NPs colloid. The yielded Au-NPs had a diameter of approximately 8 nm with a narrow size distribution.

El-Kassas et al. revealed that the formation and stabilization of Au-NPs using *Corallina officinalis* extract could be attributed to the existence of the hydroxyl functional group of polyphenols and the carbonyl group of proteins [121]. Hamida et al. extracellularly synthesized, for the first time, Ag-NPs using the novel cyanobacterial strain *Desertifilum* IPPAS B-1220 [28]. The green Ag-NPs ranged from 4.5 to 26 nm in size, were spherical, and exhibited potent anticancer and antibacterial activities. Similarly, *Nostoc Bahar M* sp. exhibited a potent reducible activity to fabricate silver nitrate into Ag-NPs at ambient temperature after 24 h under dark conditions [58]. The biogenic Ag-NPs were spherical with an average diameter of 14.9 nm and showed antiproliferative activity against colon cancer cells.

Diatoms are unicellular photosynthetic microalgae that are distinguished by hydrated amorphous silica exoskeletons of different sizes and shapes [122]. The use of live diatoms in biotechnology and their applications in ecological monitoring and biofuel production were reported in several studies [123,124]. The biosynthesis of metal NPs using live diatoms as a reducing agent has been demonstrated [125,126]. Jena et al. reported the formation of Ag-NPs by a light-dependent reaction in an aqueous cell extract of diatom *Amphora* sp. [127]. The aqueous extract of *Amphora* sp. was light yellow, indicating that only yellow pigment was extracted but not the chlorophyll. The aqueous extract was added to the silver nitrate solution for the biosynthesis of Ag-NPs. The reaction mixture started to change color from light yellow to brown within seconds and became red brown within 30 min. Ag-NPs were formed only in light conditions because no color change was observed when the reaction was conducted in dark conditions. UV–Vis spectroscopy of the Ag-NP suspension showed a peak at 413 nm. The authors reported that the increase in peak intensity at 413 nm, which was linked to the time of reaction, confirmed the rise in the number of Ag-NPs in the reaction mixture. TEM analysis revealed that Ag-NPs were polydispersed, spherical, and ranged in size from 5 to 70 nm, with an average particle size of 20–25 nm. XRD spectra revealed four intense diffraction peaks at 2θ values of 38.48°, 44°, 64.74°, and 77.4° corresponding to (111), (200), (220), and (311) planes, indicating the crystallinity of Ag-NP. These findings indicated that aqueous extract of *Amphora* sp. diatom was highly effective in reducing Ag ions to formulate scattered Ag-NPs.

#### 4.2.3. Actinomycetes

Extracellular synthesis of Au-NPs was explored using the supernatant of *Streptomyces griseoruber*, an actinomycete culture isolated from soil [128]. The development of NPs was confirmed by UV–Vis spectroscopy, which showed a peak between 520 and 550 nm. High-resolution TEM (HRTEM) analysis revealed that the formed Au-NPs were in the range of 5–50 nm and exhibited catalytic activity to degrade methylene blue.

The marine actinomycete, *Nocardiopsis alba*, isolated from mangrove soil, was utilized to produce Ag-NPs, and several bioassays were performed to evaluate the antibacterial and antiviral activities of these NPs [129]. UV–Vis spectroscopy showed the absorption peak at 420 nm, while SEM and XRD analysis revealed that the Ag-NPs were spherical and crystalline, respectively. The Ag-NPs showed antiviral activity and significant antibacterial activity against *Pseudomonas aeruginosa*, *Klebsiella pneumonia*, *Streptococcus aureus*, and *E. coli*.

#### 4.2.4. Bacteria

Bacteria, especially thermophilic bacteria, have huge potential in the extracellular green synthesis of Ag- and Au-NPs [130]. These extracellular mechanisms facilitate the production of metal NPs in an eco-friendly manner, which reduces the downstream processing of these metals.

Patil et al. investigated the effectiveness of the marine bacterium *Paracoccus haeundaensis* in the extracellular synthesis of Au-NPs and assayed the antioxidant and antiproliferative effects of the Au-NPs on both normal and cancer cells [131]. The formation of Au-NPs was confirmed by following the development of a ruby red color with a UV–Vis absorbance peak at about 535 nm. The resultant Au-NPs were spherical and had an average size of 20.93 ± 3.46 nm. The results showed no growth inhibition effect of the Au-NPs on normal cells, while the growth of cancer cells was inhibited in a concentration-dependent manner. These findings indicated that the biogenic Au-NPs were nontoxic to human cells and could therefore be used in biomedical applications.

*Bacillus brevis* (NCIM 2533) was exploited in the green synthesis of Ag-NPs [132]. The synthesized Ag-NPs, which were characterized by several spectroscopic and microscopic techniques, were spherical, had a size range of 41–68 nm, and presented with an SPR peak at 420 nm. In addition, the antibacterial effect of the Ag-NPs against multidrug-resistant pathogens, including *Salmonella typhi* and *Staphylococcus aureus*, was verified in vitro.

#### 4.2.5. Fungi

Fungi contain a plethora of biocompounds; approximately 6400 have been extracted from filamentous fungi making these organisms attractive in many applications [133]. Furthermore, these microorganisms have a potentially reduced ability to produce NPs from many bulk materials owing to their tolerance against heavy metals and potentiality to accumulate metals [134].

Molnár et al. synthesized Au-NPs using 29 different thermophilic fungi and compared the results of the extracellular fraction to those of the intracellular fraction of the fungi [65]. The fabricated Au-NPs had a size ranging between 6 and 40 nm, and the sizes vary according to the fungal strain and experimental conditions.

Another study focused on exploring the anticancer activity of Au-NPs synthesized using *Fusarium solani* [135]. Properties of the Au-NPs were observed by UV–Vis spectroscopy, FTIR, SEM, and XRD. SEM images revealed that the average diameter of the NPs was between 40 and 45 nm. These Au-NPs demonstrated dose-dependent cytotoxicity against cervical cancer cells and human breast cancer cells by inducing apoptosis pathways. The findings of this research present a safer chemotherapeutic agent with lower systemic toxicity.

#### 4.2.6. Lichens

Lichens are composite organisms that live in both obligate and beneficial symbiosis with fungi, algae, perennial trees, or cyanobacteria [34]. These organisms have been used globally in enceinte traditional medicine. Some lichens are recognized as an effective treatment for gastritis, diabetes, hemorrhoids, dysentery, dyspepsia, amenorrhea, vomiting, and respiratory tract illnesses such as pulmonary tuberculosis, throat irritation, bronchitis, and dry cough [136]. Many countries are using commercial lichen-derived pharmacological products. For example, usnic acid was used in antiseptic products in Germany (Camillen 60 Fudes spray and nail oil) and Italy (Gessato™ shaving) [137]. Icelandic lichens were used in cold remedies by the trade names of Isla-Moos^®^ (Engelhard Arzneimittel GmbH & Co. KG, Germany) and Broncholind^®^ (MCM Klosterfrau Vertriebsgesellschaft mbH, Germany). The riminophenazine was demonstrated as antimycobacterial drugs [138]. Generally, lichens contain high proportions of phenolic compounds and polysaccharides such as lichenan and isolichenan, and various secondary metabolites, including protolichesterinic acid and fumarprotocetraric acid [34,139]. These biomolecules make the lichen extracts have many biological activities such as antioxidant, antimicrobial and anticancer potencies. Moriano et al. investigated the antioxidant potency of 10 lichen species of *Parmeliaceae* spp. using oxygen radical absorbance capacity (ORAC) and 1,1-diphenyl-2-picrylhydrazyl (DPPH) radical scavenging activities and the ferric reducing antioxidant power [140]. The data exhibited that antioxidant capacities were variable between lichen species. For instance, methanolic extract of *Flavoparmelia euplecta* showed the highest ORAC value (3.30 μmol TE/mg dry extract), *Myelochroa irrugans* methanolic extract demonstrated the maximum DPPH scavenging activity (EC_50_ = 384 μg/mL), and the extract of *Hypotrachyna cirrhata* showed the highest reducing power (316 μmol of Fe2^+^ eq/g sample).

Felczykowska et al. studied the antiproliferative potency and antibacterial activity of acetonic extracts of three lichen species, namely, *Caloplaca pusilla, Protoparmeliopsis muralis*, and *Xanthoria parietina* [141]. The scholars exhibited that *P. muralis* significantly suppressed the growth of *Bacillus subtilis, Enterococcus faecalis, Staphylococcus aureus,* and *Staphylococcus epidermidis*. Moreover, *X. parietina* showed antiproliferative activity against both Hela and MCF-7 cancer cells with IC_50_ values of 8 μg/mL, and *C. pusilla* revealed the highest potency to reduce Hela, MCF-7, and PC-3 cancer cells viability with IC_50_ values of 6.57, 7.29, and 7.96 μg/mL, respectively.

Usnic acid, along with isodivaricatic acid, 5-propylresorcinol, and divaricatinic acid derived from *Protousnea poeppigii* and *Usnea florida*, showed potent antifungal activity against *Microsporum gypseum*, *Trichophyton mentagrophytes*, and *T. rubrum* [142].

Furthermore, lichen extract has been established as an efficient reducing and capping agent for NPs due to the vast abundance, rapid growth, and most importantly, environmental sustainability of these organisms [143]. The functional groups of secondary metabolites from lichen extracts are instrumental in preventing aggregation of NPs and hence improve the fabrication and stabilization of NPs [144]. Lichen-based NPs show great potential as therapeutic agents, serving as antimicrobials, antidiabetics, and antioxidants [145,146].

## 5. Lichens as Biosynthesizers for Nanoparticles

### 5.1. Metallic Nanoparticles (MNPs)

MNPs have become the most fundamental NMs in many applied and research areas due to their unique physical, chemical, and biological properties that make them promising candidates in the fields of industry, medicine, electronics, etc. [4,147]. The most frequently studied MNPs are Ag-NPs and Au-NPs due to their significant therapeutic activity against many serious diseases and their smaller-size-to-large-surface-area ratio, which enables them to be used as drug delivery systems and catalysts [8,148].

Siddiqi et al. reported that aqueous-ethanolic extract of *Usnea longissima* has the potential to fabricate silver nitrate into Ag-NPs extracellularly under laboratory conditions [136]. The lichen samples were washed, dried at 60 °C, and then crushed into fine powder [136]. Next, 10 g lichen powder was refluxed into 100 mL ethanol-distilled water (50:50) for 3 h. The samples were centrifuged to remove debris and 10 mL of supernatant was mixed with 1 mL of 0.01 M solution of AgNO_3_. The reduction process was completed after 72 h of incubation under stirring and dark conditions at room temperature. The resultant Ag-NPs were stable for weeks; however, the yield of NPs was extremely low at approximately 35%. TEM of the synthesized Ag-NPs indicated that the NPs were spherical with an average nanosize of 10.49 nm. FTIR peaks of the Ag-NPs exhibited distinct bands at 3500–3300 cm^−1^ that refer to primary amines, while bands at 1600–1500 cm^−1^ correspond to C–O stretching and amide bands (N–H). Additionally, at 1650 cm^−1^, amide I and amide II bands were reported, and at frequencies of up to 1600 cm^−1^, a COO band overlapped with the amide II band. The authors speculated that organic molecules of *U. longissima* were responsible for the reduction of silver nitrate into Ag-NPs.

The bioreduction ability of *Cetraria islandica* extract for the fabrication of silver nitrate into Ag-NPs was first studied by Yıldız et al. [139]. They reported that *C. islandica* can extracellularly fabricate Ag-NPs with diverse morphologies and sizes under different parameters such as time of exposure, the concentration of both silver nitrate and lichen extracts, and temperature. Increasing the time of exposure resulted in an increase in UV absorbance values, indicating higher production of NPs. Low AgNO_3_/lichen ratio and low temperature caused increases in absorbance values, which again indicated higher production of NPs. The authors suggested that the higher production of Ag-NPs may be due to an increase in bioreducing agents (low AgNO_3_/lichen ratio) represented by the lichen extract. It was also speculated that the average size of the Ag-NPs (5–29 nm) may be controlled by varying the silver nitrate and lichen concentrations, time of reaction, and temperature.

Khandel et al. studied the catalytic activity of *Parmotrema tinctorum* to form silver-NPs [149]. *P. tinctorum* could effectively synthesize Ag-NPs from silver nitrate in an eco-friendly manner. These NPs are distinguished by their high stability, spherical shape, and average diameter of 15.14 nm. Leela and Anchana reported for the first time the potentiality of aqueous extracts of *Parmelia perlata* and their purified fractions (secondary metabolites) to fabricate silver nitrate into Ag-NPs [150]. Briefly, the lichen extract was prepared with a cold extraction method utilizing methanol and water, in which 50 g pulverized lichen was mixed with 500 mL methanol and incubated under dark conditions on a rotary shaker for three days at ambient temperature and the same amount of lichen powder was mixed with 500 mL distilled water and boiled for 1 h at 65 °C. Each mixture was filtrated using Whatman filter paper No. 1, and the filtrates were used for the synthesis of NPs. Thin-layer chromatography (TLC), column chromatography (CC), and gas chromatography-mass spectroscopy were performed to obtain purified secondary metabolites to use in Ag-NP synthesis. Both aqueous extract of lichen and their secondary metabolites were potent reducing and stabilizing agents for the fabrication of Ag-NPs.

Aqueous extracts of *Parmotrema praesorediosum* and *Ramalina dumeticola* were also exploited for the extracellular fabrication of Ag-NPs after 72 h at room temperature [151]. Both lichen species could form Ag-NPs, but *R. dumeticola* showed the highest bioreduction activity. *R. dumeticola* induced the formation of spherical Ag-NPs with an average size of 20 nm, while those synthesized by *P. praesorediosum* were spherical with an average size of 42 nm. *P. praesorediosum* was recently reported to extracellularly synthesize Ag-NPs with a cubic structure and a nanodiameter of 19 nm [152]. Similarly, *Cetraria islandica* was an effective biosynthetic source for both Ag-NPs and Au-NPs [144]. This lichen could produce spherical silver-NPs and gold-NPs with a dominant nanosize of 6 and 19 nm, respectively, after 30 min at 80 °C. The authors suggested that oxidation of phenolic compounds was a result of the reduction process of metal ions into their nanoform.

Dasari et al. used in vitro cultures of four species of lichen, *Parmeliopsis ambigua*, *Punctelia subrudecta*, *Evernia mesomorpha*, and *Xanthoparmelia plitti* to synthesize Ag-NPs extracellularly [153]. These lichens were collected from Goolapalli, Ramakuppam Mandal, Chittoor (District), Andhra Pradesh, India. Five grams of lichen thalli was cut, washed with water, and then sterilized with 0.01% HgCl_2_. Small pieces of the thalli were then inoculated on plates of malt yeast extract medium and incubated at 28 ± 5 °C for 7–10 days before transfer to fresh culture media. Four types of mycelial mat were collected separately by filtering the cultures through Whatman No. 1 filter paper. Each type of mat was separately added to 100 mL of 1 mM silver nitrate solution and incubated for 24 h at room temperature with shaking and light conditions. The solutions were then centrifuged for 10 min at 12,000 rpm to collect the synthesized Ag-NPs. UV–Vis spectrum analysis was used to examine the reduction of Ag^+^ ions into Ag-NPs. The absorbance peak maximum was at 410–420 nm, which is typical for Ag-NPs, while the control solution (incubated without silver nitrate) did not show any peak of absorbance. The samples displayed a broad resonance (390–420 nm), suggestive of the aggregation of Ag-NPs. SEM analysis of the formed Ag-NPs disclosed their different sizes ranging between 150 and 200 nm and that the Ag-NPs were in a polydispersed mixture. FTIR analysis was conducted to identify the biomolecules present in the mycobiont mat and responsible for Ag-NPs synthesis. Briefly, samples were mixed with KBr at a ratio of 1:100, and the spectra were recorded at 1000–3500 cm^−1^. Ag-NPs synthesized by *Parmeliopsis ambigua* had an IR spectra peak at 3332 cm^−1^ that confirmed the presence of polyphenolic –OH group and peaks at 1639 and 1252 cm^−1^ that reflected the presence of amide I and carboxylic groups, respectively. Similar findings were obtained for the *Punctelia subrudecta* sample. Ag-NPs of *Evernia mesomorpha* also showed the same functional groups, –OH and –NHCO, at peaks of 3248 and 1739 cm^−1^, respectively. However, spectra of Ag-NPs synthesized by *Xanthoparmelia plittii* revealed the presence of C–N at a peak of 1015 cm^−1^ and the asymmetric mode of both the aliphatic and aromatic functional group –C–H peaking at 2923 cm^−1^. The presence of C–H stretching was confirmed by a peak at 2853 cm^−1^, and a peak at 3234 cm^−1^ corresponded to primary aliphatic amines. The presence of carbonyl group C=O from the phenols was indicated by the peak at 1656 cm^−1^, and the C–O single bonds were indicated by a peak at 1000–1200 cm^−1^, while the aromatic C–H functional group was found below 700 cm^−1^. The predicted phenols in the samples included catechin gallate, epicatechin gallate, and gallocatechin gallate. The authors speculated that polyphenolic compounds were the essential molecules in the bioreduction process of Ag-NPs.

The efficiency of aqueous extract of the lichen *Ramalina dumeticola* as a reducing and stabilizing agent for extracellular fabrication of silver nitrate into Ag-NPs was recently explored [154]. The reaction between the lichen aqueous extract (10 mL) and 30 mL of 1 mM silver nitrate solution was conducted at room temperature for 24 h. The formation of Ag-NPs was confirmed by the solution turning yellowish brown. NPs were obtained from the solution by centrifugation at 5000 rpm for 20 min and were subsequently freeze dried. UV–Vis spectral analysis, which was taken with a resolution of 2 nm at a range of 400–450 nm, monitored the formation of Ag-NPs and revealed the characteristic SPR band of Ag-NPs at approximately 433 nm. XRD analysis to define the chemical composition and crystal structure of the sample showed four peaks of 38.1°, 44.3°, 64.4°, and 77.4° at 2θ, which corresponded to the (1 1 1), (2 0 0), (2 2 0), and (3 1 1) crystallographic planes of face-centered cubic of silver, respectively. The average of crystal size was calculated by utilizing the Debye-Scherrer equation, i.e., D = (0.94λ)/(β cos θ), where D is the mean crystallite domain size, λ is the wavelength of Cu_kα_, β is the full width at half maximum (FWHM), and θ is the Bragg diffraction angle. The average size of the Ag-NP crystals was 17.1 nm. TEM revealed that Ag-NPs were polydispersed and mainly spherical with a size between 6 and 28 nm and an average diameter of 13 nm.

Rai and Gupta tested the possibility of biofabricating Ag-NPs by exploiting the reducing capacity of aqueous extracts of the lichen *Cladonia rangiferina* [145]. The lichen was collected from the Govind wildlife sanctuary in the Uttarkashi District of Uttarakhand, western Himalaya, at an altitude above 3500 m. Silver nitrate solution (45 mL of 1 mM) was mixed with 15 mL lichen aqueous extract and 2–3 drops of 0.1 M sodium hydroxide to reach an alkaline pH. After 72 h at room temperature, the reaction mixture turned yellow brown, verifying the presence of Ag-NPs. UV–Vis spectrophotometry indicated that the spectral band peak at 402 nm corresponded to the specific color change that resulted from the reduction of silver ions to Ag-NPs by secondary metabolites of the lichen. The presence of these secondary metabolites was consolidated by the detection of their functional groups by FTIR analysis. The FTIR scan taken at a range of 450–4000 cm^−1^ showed several functional groups corresponding to specific biomolecules in the extract such as polyphenols that could participate in the fabrication and stabilization of Ag-NPs. Peaks were observed in the range of 1000–4000 cm^−1^, demonstrating the presence of O–H (3400 cm^−1^), C–H (2853 cm^−1^), C=O (1742 cm^−1^), C=O (1691 cm^−1^), C=O aldehyde (1651 cm^−1^), C=C vibration (1573 cm^−1^), CH_2_, CH_3_ (1443 cm^−1^), and C–O (1273 cm^−1^). For TEM analysis, the solution was sonicated for 15 min, loaded onto a carbon-coated copper grid, and incubated under a fume hood for 30 min for the solvent to evaporate. The Ag-NPs visualized by TEM were spherical and rod shaped, with a particle size ranging from 5 to 40 nm and an average diameter of 20 nm. Similarly, different studies were reported that lichen species including *Xanthoria elegans*, *Usnea antractica*, *Leptogium puberulum*, *Cetraria islandica*, *Pseudevernia furfuracea*, *Lobaria pulmonaria*, *Heterodermia boryi* and *Parmotrema stuppeum* have potentiality to reduce silver nitrate into Ag-NPs with different shapes (bimodal and cubic) and sizes [‎^155^,^156^,^157^].

Ethanolic extract of the lichen *Parmotrema clavuliferum* was used for the biological synthesis of Ag-NPs from silver nitrate [158]. The extraction was carried out by adding 10 g dried lichen to 100 mL ethanol and incubating with shaking at 80 °C for 24 h then filtering through 25-mm pore-sized papers. The extract was added to silver nitrate solution at room temperature and the production of Ag-NPs was indicated by brown color development. The brown color appeared immediately, which verifies the high potency of *Parmotrema clavuliferum* extract as a reducing and capping agent for NPs. For further confirmation, the excitation of SPR provided by the Ag-NPs was measured spectrophotometrically at 400–450 nm. The plasmon absorption bands showed an absorbance peak at 440 nm. The DLS and zeta potential data indicated that the particle size distribution of the biogenic Ag-NPs was in the range of 80–120 nm and the particles had negative charges suggesting their stability at room temperature. TEM and SEM revealed that the Ag-NPs were spherical and approximately 106 nm in diameter. Potential biomolecules in the lichen extract were explored using FTIR spectroscopy, and broad peaks at 3264 and 1634 cm^−1^ were recorded, which correspond to O–H of phenolic compounds stretching groups and C=O of the peptide bond, respectively. These findings imply the role of phenolic compounds and in the bioreduction of silver ions.

Abdolmaleki et al. used two lichen species, *Usnea articulata* and *Ramalina sinensis*, to reduce 1 mmol of silver nitrate solution into Ag-NPs [143]. The resulting Ag-NPs were spherical with a nanosize range of 10–50 and 50–80 nm, respectively.

Recently, aqueous extracts of two novel lichen species, *Acroscyphus sphaerophoroides Lev* and *Sticta nylanderiana*, were utilized to fabricate chloroauric acid (10^−3^ M HAuCl_4_) into Au-NPs at room temperature for 12 h [159]. Physicochemical analyses confirmed the potentiality of both lichen species to generate gold-NPs. The UV–Vis-spectra of biogenic Au-NP was at 535 nm and the XRD pattern confirmed the face-centered cubic of Au-NPs. FTIR spectra of both types of Au-NPs featured bands at 3446 and 1041 cm^−1^ that relate to N–H and C–O stretching, respectively, bands at 2922 and 2849 cm^−1^ corresponding to C–H stretching, and bands at 1638 and 1456 cm^−1^ that relate to the amide and carboxylate groups, respectively, in the amino acid residues of the biomolecules. The authors speculated that the presence of these functional groups might help prevent agglomeration of the NPs. Moreover, TEM revealed that *A. sphaerophoroides*-mediated Au-NPs are multiply twinned quasi-spherical and prismatic with a size range between 5 and 35 nm, while *S. nylanderiana*-mediated Au-NPs were exclusively multiply twinned with a nanosize range of 20–50 nm.

An extract of the lichen *Parmelia sulcate* was used to synthesize Au-NPs; for its preparation, 90 mL of a 1 mM HAuCl_4_ solution (to provide Au^3+^) and 10 mL of the 5% *Parmelia sulcata* extract were heated to 60 °C and kept on a magnetic stirrer for 20 min [160]. The color change of the reaction mixture from yellow to purple was monitored to observe the formation of Au-NPs then the solution was dried in an oven (70 °C) for 48 h to obtain powdered particles. The UV–Vis spectrum from 300–700 nm confirmed the reduction of Au^3+^ to gold NPs (Au^0^). The peak observed at 540 nm represents the SPR, verifying the formation of the Au-NPs. The XRD spectrum had peaks at 38.3°, 44.6°, 64.7°, and 77.7°, which revealed the crystalline feature of gold and the face-centered cubic particles depending on the angular positions of the Bragg peak. SEM and TEM demonstrated that the particles had an average size of 54 nm and were spherical. TEM with energy-dispersive spectroscopy (EDS) was utilized to determine the elemental composition. The EDS pattern displayed a strong signal for the gold peak, indicating successful fabrication of Au-NPs. FTIR spectra were measured over a range of 400–4000 cm^−1^ to follow the reaction between the *Parmelia sulcata* extract and chloroauric acid. Biomolecules in the lichen extract were confirmed to interact with Au-NPs. The peaks at 3443 cm^−1^ corresponded to the O–H strong stretch of the alcohol, while a peak at 1640 cm^−1^ corresponded to the C=C of the alkenes, and the peaks at 1544 and 1384 cm^−1^ were related to the N–H and N–O bending and stretching of the amide and nitro groups, respectively. The peaks at 1272 and 1206 cm^−1^ were related to the C–O stretch of esters. The presence of proteins in the solution, indicated by the amide and nitro group peaks, might contribute to the stabilization of the newly formed NPs. DLS and zeta potential analyses revealed that the average size of the hydrodynamic diameter of the Au-NPs was 54.14 nm and their charge was negative (−18.4). This indicates the existence of lichen biomolecules surrounding the Au-NPs, which provide stability to the Au-NPs.

Devasena et al. used the Soxhlet extraction method to obtain the lichen extract to use in magnesium nanoparticles (Mg-NPs) synthesis [161]. They reported that *Cladonia rangiferina* has the ability to reduce magnesium sulfate into Mg-NPs extracellularly. UV–Vis spectroscopy analysis revealed that the absorption peak of Mg-NPs was at 262 nm, while the DLS technique revealed that Mg-NPs have an average hydrodynamic diameter of 23 nm.

*Protoparmeliopsis muralis* was first used for the synthesis of different metallic and metal oxide NPs (MONPs) by Alavi et al., who utilized the aqueous extract of this lichen to extracellularly fabricate silver-NPs and copper-NPs under dark and stirred conditions for 24 h at ambient temperature [162]. Synthesis of the MNPs was confirmed by UV–Vis spectroscopy, TEM, SEM, EDAX, XRD, and FTIR analyses. The resulting data demonstrated that the maximum absorbance peak for Ag-NPs and Cu-NPs was 378 and 567 nm, respectively, and that the MNPs were spherical with an average nanosize of 33.49 ± 22.91 and 253.97 ± 57.2 nm, respectively. EDAX data demonstrated that Ag- and Cu-NPs were present in the sample at 87.72 and 26.42%, respectively. Furthermore, the 2θ degree values of both Ag- and Cu-NPs were 35.5°, 43.6°, 65.6°, and 72.1°, and 35.9°, 39.6°, 44.3°, 54.3°, and 57.2°, respectively, indicating the crystallinity of these NPs. Based on FTIR data, there were three dominant functional groups, C=C, S=O, and C-Br, in all samples (lichen extract, Ag- and Cu-NPs, and metal oxides NPs). However, O–H bond bending corresponding to secondary metabolites such as phenol was observed for the MNPs, suggesting that these secondary metabolites may act as a potential reducing and stabilizing agent during the synthesis process of both Ag- and Cu-NPs. To prove this hypothesis, the authors analyzed the total phenol, flavonoid, flavanol, and tannin contents (TPC, TFC, TFLC, and TTC, respectively) of the samples via Folin-Ciocaltue assay. The Ag-NP solution contained higher amounts of TPC, TFC, TFLC, and TTC, compared with the lichen extract and other MNPs and MONPs. The same study also screened the effect of time exposure (24, 48, 72, and 96 h) on the biosynthesis process of MNPs and demonstrated that the concentration of MNPs increased as the time of exposure increased. The authors noted that the synthesis process of MNPs using lichen aqueous extract was slower than that using plant watery extract suggesting the cause of the slow reaction is the lower reducing capacity of lichens (Table 1).

### 5.2. Metal Oxide Nanoparticles (MONPs)

MONPs are one of the widest used nanomaterials due to their unique properties including high stability, porosity, and easy functionalization with different molecules because of their negative charge; these properties mean MONPs are particularly suited to biomedical applications [170].

Alavi et al. utilized the aqueous extract of *Protoparmeliopsis muralis* to biosynthesize three different types of MONPs—ferric oxide, zinc oxide, and titanium oxide (Fe_3_O_4_, ZnO, and TiO_2_, respectively) NPs [162]. Briefly, lichen samples collected from Kane Gonabad Mountains were washed with distilled water, air dried for six days, then crushed into a fine powder, and boiled with 250 mL distilled water at 90 °C for 30 min. The mixture was filtered through Whatman filter paper No. 40 and 10 mL of the filtrate, was mixed with 50 mL TiO(OH)_2_ or Zn(NO_3_)_2_·6H_2_O (0.01, and 0.001 M concentrations, respectively), and incubated for 24 h with stirring. For fabrication of Fe_3_O_4_ NPs, the same amount of lichen extract was added to flasks containing FeCl_3_·6H_2_O (0.2 M) and FeCl_2_·4H_2_O (0.001, 0.01, and 0.1 M), and the pH was adjusted to 8 by adding 0.1 M NaOH solution. Mixtures were kept under stirred conditions for 24 h at room temperature. The resultant NPs were collected by centrifugation at 4000 rpm for 30 min, washed, and dried at 70 °C for 8 h. Physicochemical analyses showed that the UV-spectra peaks of Fe_3_O_4_, ZnO, and TiO_2_ NPs were 216, 328, and 283 nm, respectively. TEM and SEM images demonstrated that all the MONPs were spherical with an average nanodiameter of 307 ± 154 (Fe_3_O_4_ NPs), 133.32 ± 35.33 (TiO_2_ NPs), and 178.06 ± 49.97 nm (ZnO NPs). The presence of Fe (84.07%), Ti (66.41%), and Zn (25.61%) in the Fe_3_O_4_, ZnO, and TiO_2_ NPs samples was detected by EDAX analysis. XRD and FTIR analyses proved that these MONPs had nanocrystal structures and were coated with organic molecules such as secondary metabolites (phenols, O–H), which have a significant role in reducing and stabilizing NPs.

The hydrolytic capacity of aqueous extracts of a new strain of lichen, *Ramalina sinensis*, was recently reported to extracellularly fabricate ferric chloride salts into iron oxide NPs [165]. The UV-spectra curve of the NP samples appeared in the range of 280–320 nm, indicating the formation of magnetic iron oxide NPs. The XRD pattern of the biosynthesized iron oxide NPs showed distinct diffraction peaks of 30.5°, 36.1°, 43.3°, 53.9°, 57.5°, and 63.3° at 2θ, indicating the cubical nanocrystalline structure of iron oxide NPs. Furthermore, FTIR analysis demonstrated that π-electrons of carbonyl groups of flavonoid and phenolic compounds of *R. sinensis* were responsible for the reduction of iron ions into their nanoforms. Field emission scanning electron microscopy (FESEM) revealed that the particle size of the iron oxide NPs was between 31.74 and 53.91 nm and that pores existed in the iron oxide NPs structure. EDX analysis showed that Fe and O elements were the main constituents in the iron oxide nanostructure.

Similarly, Arjaghi et al. performed extracellular reduction of ferric chloride salts (FeCl_2_·4H_2_O and FeCl_3_·6H_2_O) into Fe_3_O_4_ NPs by utilizing *R. sinensis* [163]. Sharp absorption peaks were observed between 300 and 350 nm owing to the interaction between the chemicals, and tensile vibration resulted from the formation of a new bond between iron and oxygen and the synthesis of Fe_3_O_4_ NPs. The authors hypothesize that the existence of biomolecules in *R. sinensis* might prevent the agglomeration of NPs, that polysaccharide sulfate acts as a potent reducing agent, and that sulfate groups have significant roles in the extracellular synthesis of iron oxide NPs by oxidizing the aldehyde group into carboxylic acids. XRD and SEM data revealed that Fe_3_O_4_ NPs were nanocrystalline and 20–40 nm in size.

ZnO-NPs were biologically synthesized by Koca et al. using *Ramalina fraxinea* extract [169]. Lichen samples were carefully washed, dried in a 70 °C oven overnight, and extracted in water by heating (80 °C) for 1 h, and the resulting extract was filtered through Whatman No 1 filter paper. For the synthesis of ZnO-NPs, 100 mL filtered extract was added to 5 g Zn(NO_3_)_2_·6H_2_O and incubated at 60 °C with continuous stirring until the color changed, indicating the formation of NPs. The solution was then heated at 400 °C for approximately 2 h to obtain a fine powder of ZnO-NPs. The characteristic SPR band of the ZnO-NPs was determined by UV analysis at a wavelength range between 200 and 900 nm. Peaks were observed at 269 nm, which related to *Ramalina fraxinea* extracts, and 330 nm, suggesting synthesis of ZnO NPs was successful. FTIR analysis of the ZnO-NPs revealed O–H (alcohol) band vibrations at 3128 and 1398 cm^−1^ and stretching bands at 1620 and 1575 cm^−1^ that were correlated to the alkenes (C=C), while absorption peaks at 1480 cm^−1^ were for alkanes (C–H). Bands at 1379 and 1335 cm^−1^ related to O–H (alcohol and phenol), and the presence of amine groups (C–N) was observed at 1192 cm^−1^. The peak at 1295 cm^−1^ represented the aromatic ester (C–O) and aromatic amine groups (C–N), while the bands at 1121 and 1087 cm^−1^ were identified as amine (C–N) and aliphatic ether (C–O) groups. Stretching bands at 1038 and 960 cm^−1^ were allotted to ether (C–O) and alkene (C–H), respectively. The bands at 871, 842, 773, 671, and 605 cm^−1^ confirmed the presence of halo compounds (C–Cl), and the peaks observed at 407, 444, and 535 cm^−1^ were assigned to Zn-O (metal-oxygen) vibration. In conclusion, FTIR analysis disclosed that functional groups in the extract of *Ramalina fraxinea* are crucial for the synthesis of ZnO-NPs. XDR spectra at 2θ showed a group of diffraction peaks of 31.7°, 34.4°, 36.2°, 47.5°, 56.5°, 62.8°, 66.4°, 67.9°, 69.1°, 72.5°, and 77.1° indicative of the crystal planes of (1 0 0), (0 0 2), (1 0 1), (1 0 2), (1 1 0), (1 0 3), (2 0 0), (1 1 2), (2 0 1), (0 0 4), (2 0 2), and (1 0 4), respectively. Collectively, the XDR analysis demonstrated that the *Ramalina fraxinea* extract delivered ZnO-NPs with a characteristic hexagonal and crystalline structure. The biogenic ZnO-NPs were spherical with a size of around 21 nm, as evidenced by SEM and FESEM imaging. The authors reported the existence of aggregation of NPs that resulted from the impact of Van der Waals forces between NPs (Table 1).

### 5.3. Other Nanomaterials

Abdullah et al. introduced the green synthesis method of ZnO@TiO_2_@SiO_2_ and Fe_3_O_4_@SiO_2_ nanocomposites (NCs) utilizing the novel lichen species *Lecanora muralis* [164]. Lichen specimens were collected from Grdmandil mountain and the chemical compositions of the rock-inhibiting lichen samples were analyzed via XRD assay. The samples comprised quartz, hematite, magnetite, and maghemite Q. Similarly, biomolecules inside the lichen cell extract were determined using gas chromatography-mass spectroscopy, and there were a variety of secondary metabolites present that have many medical applications through their antioxidant, anticancer, analgesic, and antipyretic activities. To synthesize ZnO@TiO_2_@SiO_2_, 2 g of *L. muralis* (LM) was mixed with 30 mL distilled water and the mixture was boiled at 80 °C for 1 h then filtrated. Next, 20 mL LM filtrate was mixed with 0.5 g ZnCl_2_, 1.5 g TiO(OH)_2_ (titanyl hydroxide), and 2.5 g Na_2_SiO_3_ at pH 8 and 80 °C for 5 h under stirring conditions. Similarly, Fe_3_O_4_@SiO_2_ NCs were formed by mixing 20 mL LM filtrate and 2 g Na_2_SiO_3_ with 0.7 g FeCl_2_ and 1.2 g FeCl_3_ at pH 9 and 80 °C for 5 h under stirring conditions. After the incubation period, NP precipitates were filtrated, washed with hot distilled water to discard any impurities, and then dried. Physical and chemical analyses of the ZnO@TiO_2_@SiO_2_ and Fe_3_O_4_@SiO_2_ NCs showed that *L. muralis* has the potential to fabricate NCs from their bulk materials. XRD demonstrated that the biosynthesis of ZnO@TiO_2_@SiO_2_ and Fe_3_O_4_@SiO_2_ NCs generates crystallinity nanoforms of 55 and 53 nm, respectively. Furthermore, the authors reported that Fe_3_O_4_ NPs coated the surface of the silica oxide nanoparticles. SEM micrographs of ZnO@TiO_2_@SiO_2_ and Fe_3_O_4_@SiO_2_ NCs revealed that these NCs were spherical and had a nanosize range of 55–90 and 50–85 nm, respectively. Some agglomeration was observed by SEM in both types of NC. EDX and elemental mapping showed that ZnO@TiO_2_@SiO_2_ NC was synthesized from Zn, O, Ti, and Si with no further elements, indicating the purity of the formed nanostructure; however, the compositional elements of Fe_3_O_4_@SiO_2_ NCs involving Fe, O, and Si indicated the binding of Fe_3_O_4_ NPs on the surface of SiO_2_ NPs (Table 1).

Bimetallic NPs (Au–Ag NPs) were extracellularly synthesized using *Cetraria islandica* [144]. In brief, 1 mL lichen extract was mixed with 10 mL of 1.5 mm HAuCl_4_ and AgNO_3_ solutions and 0.5 m NaOH solution (pH 10) and incubated for 30 min at 80 °C under continuous mixing and stirring conditions. The reaction was repeated with different molar ratios of the Ag and Au solutions (1:1, 1:2, and 2:1), and the resulting bimetallic NPs are defined as Ag50Au50, Ag33Au67, and Ag67Au33, respectively. Only one absorption band appeared between the SPR of both monometallic Au- and Ag-NPs, and as Au content increased, the absorbance band redshifted. UV absorbance peaks of Ag50Au50, Ag33Au67, and Ag67Au33 were 412, 519, and 523 nm, respectively. This finding implied that bimetallic NPs may have only one alloy. FTIR spectra peaks for Ag33Au67 were at 1383 cm^−1^, which relate to C=O of carboxylic acid and methyl interactions in large, branched molecules that play an important role in stabilizing and capping NPs. TEM images of Ag33Au67 bimetallic NPs showed that these NPs were spherical and polygonal with nanosizes of 6 and 21 nm, respectively. The narrow particle size indicates a large active surface area for catalytic activity.

**Figure 4:**
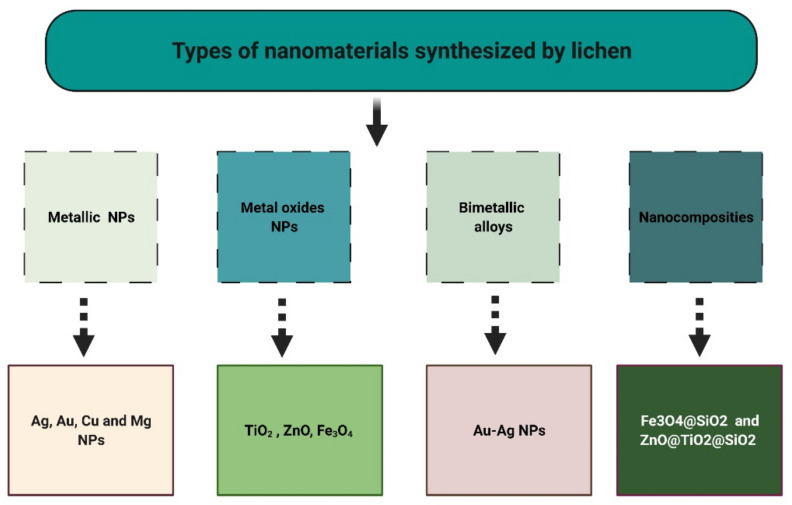
Types of nanoparticles (NPs) synthesized by lichen species.

Esmaeili and Rajaee explored an eco-friendly synthesis method using lichen usnic acid as a nanoparticle mediator to produce nanohyaluronic acid [146]. In brief, usnic acid (UAL) was extracted from *Aspicilia lichens* with acetone using a Soxhlet apparatus for 8 h. The FTIR spectrum of purified UAL was similar to the standard usnic acid spectrum. The stretching of O–H in the Ar–OH intramolecular hydrogen bond showed strong bands at 3421 and 3371 cm^−1^. Additionally, (–CH_3_) of the alkane groups in UAL had peaks at 2925 and 2854 cm^−1^ caused by stretching of the C–H bond. The presence of the C=C group in UAL was indicated by a peak at 1658 cm^−1^, which is due to the presence of the aryl group. The aromatic methyl ketone at 1625 cm^−1^ is related to the hydrogen bonds. Similarly, the conjugate cyclic ketone group is confirmed by a peak at 1739 cm^−1^. UAL also showed hydroxyl phenolic signals at 3095 cm^−1^, which is likely due to the stretching of a symmetrical or nonsymmetrical group C–O–C that bonded to an aryl-alkyl-ester at 1265 and 1074 cm^−1^. SEM micrographs demonstrated that UAL NPs were spherical with a mean size of 29–89 nm and no agglomeration. For the preparation of nanohyaluronic acid, 2 g hyaluronic acid (produced by mixing *Bifidobacterium* sp. and the solution of UAL extract) was added to 50 mL distilled water and 200 mL of a UAL solution with acetone and methanol (5:2) at 50 °C for 48 h with stirring. The NPs were then collected by centrifugation at 12,000 rpm for 30 min. SEM micrographs showed that hyaluronic NPs have an average nanosize of 55 nm. FTIR spectra of nanohyaluronic acid showed that the usnic acid extracted from *Aspicilia* sp. has strong redox activity that enables these compounds to reduce the hyaluronic acid into their nanoform (Figure 4).

## 6. Prospective Applications of Lichen-Based Nanoparticles

### 6.1. Antimicrobial Activity

Khandel et al. studied the inhibitory activity of Ag-NPs synthesized by *Parmotrema tinctorum* and the activity of silver nitrate and lichen extract against five pathogenic bacteria—*Pseudomonas aeruginosa*, *Staphylococcus aureus*, *Escherichia coli*, *Bacillus subtilis*, and *Klebsiella pneumoniae*—for 24 h at 35 °C using the agar well diffusion method [149]. Ag-NP (10, 30, and 50 µL) was the most potent antibacterial agent, causing greater inhibition of bacterial growth compared with silver nitrate and lichen extract. Ag-NPs suppressed the growth of both Gram-negative and Gram-positive bacteria at the three concentrations tested. At the highest concentration of Ag-NPs (50 µL), the greatest inhibition zone (IZ) was detected against *P. aeruginosa* (17 ± 0.50 mm), *K. pneumoniae* (14 ± 0.10 mm), and *E. coli* (11 ± 0.10 mm), while the lowest IZ was observed against *B. subtilis* (8 ± 0.30 mm) and *S. aureus* (7 ± 0.30 mm). The authors reported that Ag-NPs were more effective at inhibiting Gram-negative bacteria than Gram-positive bacteria due to the difference in the cell-wall structure of the bacteria; Gram-positive bacteria have a thicker cell wall than Gram-negative bacteria, and hence, penetration of their cell wall is difficult. Furthermore, the authors conclude that the mode of action of Ag-NPs against bacteria is via their ability to change the membrane structure and permeability, leading to bacterial death.

Iron oxide-NPs (0.075–0.00046875 mg/mL) bioformed by *Ramalina sinensis* significantly inhibited the bacterial growth of both *P. aeruginosa* and *S. aureus* after incubation for 24 h at 37 °C [165]. Iron oxide-NPs at 0.075 mg/mL exhibited the highest antibacterial activity, while 0.0075 and 0.000234375 mg/mL of iron oxide-NPs were the lowest inhibitory concentrations of NPs against both *P. aeruginosa* and *S. aureus*, respectively. The antibacterial activity of iron oxide-NPs was almost equivalent to that of tetracycline. The authors suggested that the expected killing mechanism of NPs against bacteria may be related to the electrostatic activity between iron oxide-NPs and the bacterial membrane. This interaction might result in the release of the iron ions by the NPs, and these ions can then interact with the thiol group on membrane proteins, causing bacterial membrane oxidation, subsequent stimulation of reactive oxygen species (ROS), loss of membrane permeability, disruption of cell membrane respiration, and ultimately, bacterial death.

Abdullah et al. studied the antibacterial and antifungal activities of both ZnO@TiO_2_@SiO_2_ and Fe_3_O_4_@SiO_2_ NCs synthesized by *Lecanora muralis* against *S. aureus*, *E. coli*, *Pseudomonas* sp., and five species of fungi, i.e., *Candida albicans*, *Candida* spp., *Aspergillus flavus*, *Aspergillus niger*, and *Aspergillus terrus*, utilizing both disk diffusion and agar well diffusion assays and compared the results with those of lichen extract alone [164]. Both NCs showed higher inhibitory activity than lichen extract alone against bacterial and fungal species, with the exception of the three species of the genus *Aspergillus* (zero inhibition zone). Fe_3_O_4_@SiO_2_ exhibited the highest bioactivity among the treatments, suggesting more bioactive molecules were precipitated on Fe_3_O_4_@SiO_2_ NCs than on ZnO@TiO_2_@SiO_2_ NCs. The authors noted that the increased antioxidant molecules adsorbed on Fe_3_O_4_@SiO_2_ NCs contributed to the long-term stabilization of NCs against decomposition and deformation conditions.

Lichen ethanolic extract (*Parmotrema clavuliferum*) and the corresponding lichen-synthesized Ag-NPs were investigated as an antibacterial treatment in a recent study by Alqahtani et al. [158]. Biogenic Ag-NPs showed a significant inhibitory effect against *P. aeruginosa* (11.5 ± 0.9 mm), *Streptococcus faecalis* (7.6 ± 1.7 mm), *B. subtilis* (8.1 ± 1.5 mm), and *S. aureus* (8.1 ± 1.5 mm). However, the ethanolic extract of the lichen caused had the highest zone of inhibition (19.8 ± 0.9 mm) against *B. subtilis* and the lowest zone of inhibition (3.6 ± 0.9 mm) against *S. aureus*. Furthermore, *S. faecalis* and *P. aeruginosa* showed inhibition zones of 15.5 ± 1.6 and 13.8 ± 0.9 mm, respectively, after spiking with lichen extract. The authors suggested that Ag-NPs could have this bactericidal effect due to one or more of the following actions: Ag-NPs may induce cell lysis, hinder transduction, change membrane permeability, or destroy the bacterial genome through DNA fragmentation.

Ag-NPs produced using four lichens, *Parmeliopsis ambigua*, *Punctelia subrudecta*, *Evernia mesomorpha*, and *Xanthoparmelia plitti*, were screened for antibacterial activity against several Gram-negative and Gram-positive bacteria, including *Pseudomonas aeruginosa*, *Escherichia coli, Proteus vulgaricus, Staphylococcus aureus*, *Streptococcus pneumoniae*, and *Bacillus subtilis* [153]. The disk diffusion method was used for screening with 0.02 mg of the produced Ag-NPs. *Pseudomonas putida* was the most susceptible to Ag-NPs synthesized by *X. plitti* (2.3 cm), followed by *Pseudomonas aeruginosa* with Ag-NPs synthesized by *E. mesomorpha* extract (1.9 cm) and *Bacillus subtilis*, which was the least susceptible with Ag-NPs synthesized by *X. plitti* extract (1.3 cm).

The antibacterial activity of Ag-NPs synthesized using an aqueous extract of *Ramalina dumeticola* were examined against four Gram-positive pathogenic bacteria (*Staphylococcus epidermidis*, methicillin-resistant *Staphylococcus aureus* (MRSA), *Bacillus subtilis*, and *Streptococcus faecalis*) and four Gram-negative strains (*Proteus vulgaris*, *Pseudomonas aeruginosa*, *Serratia marcescens*, and *Salmonella typhi*) by applying the disk diffusion method [154]. Gentamycin (30 μg disks) was used as a positive control, and the negative control was sterile distilled water. A total of 10 microliters of 100 μg/mL of the Ag-NPs solution was applied to the sterile disks on agar plates, and inhibition zones (IZs) were measured after incubation for 18–24 h at 37 °C. The results demonstrated the potential of the Ag-NPs as a bactericidal factor. The Ag-NPs were effective against both types of bacteria but showed more efficacy towards Gram-negative bacteria than Gram-positive bacteria. The largest IZ was observed in *Proteus vulgaris* (10.5 ± 0.7 mm), followed by *Pseudomonas aeruginosa* (9.5 ± 3.5 mm) and MRSA (9.5 ± 0.7 mm). The Ag-NPs were less effective in *Salmonella typhi* (IZ diameter of 8.5 ± 2.1 mm) and *Serratia marcescens* (7.5 ± 0.7 mm). *Bacillus subtilis* and *Streptococcus faecalis* had identical IZ (7.5 ± 0.0 mm), while the least inhibitory effect of the Ag-NPs was observed on *Staphylococcus epidermidis* with an IZ of 7 ± 0.0 mm. The same concentration of aqueous extract (100 μg/mL) resulted in a lower inhibition zone diameter of 6 mm against all tested microbes compared with the IZ diameters produced with Ag-NPs. This suggested that the Ag-NPs have higher antibacterial activity than the lichen extract. The authors hypothesize that the increased susceptibility of Gram-negative bacteria to Ag-NPs compared with Gram-positive bacteria is likely due to the thinner peptidoglycan layer of Gram-negative bacteria, which provides the Ag-NPs with better anchoring and penetration of the cell wall.

Ag-NPs synthesized by Usnea longissima were evaluated for antimicrobial potency against six-Gram positive bacteria (*Staphylococcus aureus*, *Streptococcus mutans*, *Streptococcus pyogenes*, *Streptococcus viridans*, *Corynebacterium diphtheriae*, and *Corynebacterium xerosis*) and three Gram-negative bacteria (*Escherichia coli*, *Klebsiella pneumoniae*, and *Pseudomonas aeruginosa*) by agar well diffusion method [136]. The bacteria were incubated with Ag-NPs for 24 h at 37 °C. A negative control (DMSO) and positive controls (ciprofloxacin (5 μg/disk) for Gram-positive bacteria and Gentamicin (10 μg/disk) for Gram-negative strains) were used to compare the inhibitory activity of NPs. The Ag-NPs displayed the highest antibacterial efficiency against *E. coli* and *K. pneumoniae* with IZ diameters of 20.8 ± 0.02 and 16 ± 0.31 mm, respectively. In contrast, *S. mutans* (6.5 ± 0.89 mm), *C. diphtheriae* (6.2 ± 0.37 mm), and *P. aeruginosa* (7 ± 0.31 mm) were not affected by the Ag-NPs. The Ag-NPs were suggested to have a low antibacterial effect on the basis that the antibacterial effect can be amplified by reducing the NP size and hence increasing the surface area. As the surface area of the Ag-NPs increases, contact with microorganisms improves, which mediates penetration of the particles into the bacterial cell membrane or attachment to the bacterial surface. When silver ions reach the bacterial cytoplasm, they can denature the ribosome, thus directing the suppression of cell enzymes and proteins. Consequently, the metabolic function of the bacterial cell will be disrupted and the cell will undergo apoptosis. The authors reported that the lethal effect of Ag-NPs against bacteria can be achieved by different mechanisms including (i) interfering with cell wall, (ii) suppression of protein synthesis, and (iii) disruption of transcription and primary metabolic processes.

Kumar et al. studied the synergistic antibacterial effect of the extracts of two lichens, *Parmotrema pseudotinctorum* and *Ramalina hossei*, combined with chemically synthesized Ag-NPs, against several strains of Gram-positive and Gram-negative bacteria known to cause food poisoning [171]. The tested strains *Staphylococcus aureus*, *Bacillus cereus*, *Escherichia coli*, and *Salmonella typhi* were treated with the lichen extracts and Ag-NPs individually and with a combination of both, utilizing the agar well diffusion method. On Muller-Hinton agar plates, bacterial broth cultures (10^8^ cells/mL) were swabbed then wells of 6-mm diameter were loaded as follows: lichen extracts (10 mg/mL in DMSO), Ag-NPs (1 mg/mL in DMSO), standard (chloramphenicol, 1 mg/mL), a combination of lichen extract and Ag-NPs (1:1 ratio), and control (DMSO). The plates (two replicates of each) were incubated at 37 °C for 24 h and the IZs were measured and the mean value was calculated for each sample. According to the IZs, the lichen extracts were more effective than the Ag-NPs alone on most plates. The Ag-NPs were more effective against Gram-negative bacteria than Gram-positive bacteria. However, the combination of the lichen extracts and Ag-NPs showed more bacterial inhibition than that of the extract alone or the NPs alone. After exposure to the combined treatment, *S. typhi* had an IZ of 2.8 cm, followed by *E. coli* (2.6 cm), *B*. *cereus* (2.1 cm), and *S. aureus* (1.9 cm). The enhanced antibacterial activity of the combined treatment might be attributed to the presence of effective secondary metabolites in the lichen extracts, and also the smaller-size-to-large-surface-area ratio of Ag-NPs. In the lichen extracts experiments, Gram-positive bacteria were more affected than Gram-negative bacteria. However, in the combined treatment assays, the antibacterial activity was more pronounced against Gram-negative bacteria. The authors attributed this to Gram-negative bacteria being naturally more resistant due to their thick outer membrane that prevents harmful substances from entering the cell. This barrier comprises an exterior lipopolysaccharide layer and a thin layer of peptidoglycan at the interior. TEM imaging confirmed that Ag-NPs can be effective bactericidal agents by rupturing the bacterial membrane even at low concentrations.

### 6.2. Antioxidants

The antioxidant activity of biomatrix loaded with Au-NPs synthesized by *Acroscyphus sphaerophoroides* and *Sticta nylanderiana* was screened by Debnath et al. using a modified diphenylpicrylhydrazyl (DPPH) method. Powdered samples of 2 and 5 mg were treated in two separate test tubes with 3 mL of 100 M methanolic solution of DPPH. The surface reaction for both mixtures was amplified by sonicating them in the dark. To confirm time-dependent DPPH scavenging, centrifugation was performed and the absorbance of the supernatants over time was measured at 517 nm with DPPH as a reference and a gap of 15, 30, 45, and 60 min. Measurement of the scavenging potential (SC_50_) of biomatrix-loaded Au-NPs synthesized by *A. sphaerophoroides* and *S. nylanderiana* is achieved via a similar process, where absorbances are documented at 30 min after administering 1, 1.5, 2, 2.5, 3 mg and 1, 3, 5, 7, 10 mg of the samples, respectively. The concentrations of gold-NPs synthesized by *A. sphaerophoroides* and *S. nylanderiana* responsible for scavenging of 50% of DPPH (SC_50_) were 1.66 and 4.48 mg, respectively, suggesting biogenic gold-NPs were potent antioxidant agent [159].

An extract of the lichen *Parmelia sulcata* was exploited for the biological formulation of Au-NPs [160], and the resulting Au-NPs and *P. sulcata* extract were tested for their free radical scavenging potential in antioxidant bioassays involving DPPH and hydrogen peroxide. For the DPPH method, 2.96 mL of 0.1 mM solution of DPPH was added to 0.4 mL of the extract or Au-NPs at different concentrations (250, 500, 750, and 1000 μg/mL) and incubated under dark conditions at ambient temperature for 30 min. The absorbance was recorded at 517 nm and used to calculate the percentage inhibition of scavenging potential. For the hydrogen peroxide scavenging test, 40 mM H_2_O_2_ solution was prepared in phosphate buffer at pH 7.4 and then several concentrations (250, 500, 750, 1000 μg/mL) of extracts and Au-NPs were added and incubated for 10 min at room temperature. The absorbance was measured at 230 nm and was subsequently used to determine the percentage of inhibition. The outcomes of these bioassays consolidated the ability of the lichen extract and Au-NPs to scavenge free radicals; the IC_50_ values of DPPH were 1020 and 815 μg/mL and the IC_50_ values of H_2_O_2_ were 694 and 510 μg/mL, respectively. These results indicated that the Au-NPs had greater potential for free radical scavenging (FRS) compared with the lichen extract. In addition, the FRS activity of both lichen extract and Au-NPs appears to be concentration dependent.

### 6.3. Other Applications

A recent study used ZnO-NPs biosynthesized by *Ramalina fraxinea* extract as a cytotoxic agent for human neuroblastoma cells [169]. The study focused on evaluating the neurotoxicity and neuroprotective effect of lichen-synthesized ZnO-NPs against SHSY-5Y human neuroblastoma cells. Several concentrations of ZnO-NPs were prepared to identify the cytotoxic doses of these NPs. A concentration of 25 µg/mL ZnO-NPs significantly increased the cell viability (*p* < 0.05) when compared with the control group. However, a lower concentration (5 µg/mL) of ZnO-NPs did not affect SHSY-5Y cells. ZnO-NPs at 50, 100, 200, and 400 µg/mL caused a marked reduction (*p* < 0.001) in cell viability, compared with those of the control group. To estimate the neuroprotective effect of ZnO-NPs, the authors exploited the ability of hydrogen peroxide to induce apoptosis of SHSY-5Y cells via oxidative stress; for this purpose, 300 µM H_2_O_2_ was used to treat the cells. This treatment resulted in a significant reduction (*p* < 0.001) of cell viability compared with the control group. ZnO-NPs (at all tested doses) did not increase H_2_O_2_-induced death of the SHSY-5Y cells. Moreover, the higher doses (100, 200, and 400 µg/mL) of ZnO-NPs markedly reduced (*p* < 0.001) the cell viability compared with the H_2_O_2_ group. In summary, ZnO-NPs at high doses (≥50 µg/mL) can induce neurotoxicity in SHSY-5Y neuroblastoma cells but provide neuroprotection against the neurotoxic effect of hydrogen peroxide at a low to moderate doses (25 µg/mL).

Iron oxide-NPs fabricated by *Ramalina sinensis* were able to remove lead and cadmium (82 and 77%, respectively) from aqueous solution at an initial concentration of 50 mg/L and with pH in the range of 5–4, indicating the potential of these NPs to be heavy metal eliminators [163].

Çıplak et al. conducted the first study on the catalytic activity of biogenic monometallic NPs (Ag- and Au-NPs) and bimetallic NPs (Au-Ag NPs) synthesized by *Cetraria islandica* [144]. Bimetallic NPs showed higher catalytic activity than monometallic NPs for the reduction of nitrophenols (4-nitrophenol; 4-NP) to aminophenols (4-aminophenol; 4-AP) with sodium borohydride (NaBH_4_). The higher catalytic performance of Au-Ag NPs might be attributed to the higher ionization potential of Au (9.22 eV) than Ag (7.58 eV), which causes electronic charge transfer from Ag to Au and results in an increase in the electron density on the NP surface. Similarly, Au-NPs exhibited better catalytic potentiality than the Ag-NPs.

The reducing power, hydrogen peroxide scavenging ability, and antidiabetic activities of Ag-NPs synthesized by both *Parmelia perlata* aqueous extract and their purified glycoside and alkaloid fractions were screened by Leela and Anchana [150]. Biogenic Ag-NPs generated from lichen fraction biomolecules have significant antidiabetic potential, reducing power, and free radical scavenging ability, compared with the Ag-NPs fabricated by lichen aqueous extract. The antidiabetic properties of the biogenic Ag-NPs were tested using an alpha-amylase inhibition assay and the percentage inhibition of alpha-amylase was 11.11% for Ag-NPs synthesized by lichen aqueous extract, and 51.85 and 29.62% for the Ag-NPs fabricated by the glycoside fraction and alkaloid fraction, respectively. This indicated that glycoside-mediated-Ag-NPs exhibited the strongest antidiabetic activity. The authors suggest that these biogenic Ag-NPs may lead to improvements in type 2 diabetic disease. Furthermore, the reducing activity of the same Ag-NPs was explored in a reducing power assay in which Ag-NPs interact with potassium ferricyanide (Fe^3+^), leading to the generation of potassium ferricyanide (Fe^2+^), which then reacts with ferric chloride to form a ferric-ferrous complex that is readily detected by UV spectrophotometer. Glycoside-mediated-Ag-NP had the greatest reducing activity among the three types of Ag-NPs (absorbance of 0.771, compared with 0.639 and 0.4 for Ag-NPs fabricated utilizing lichen aqueous extract and the alkaloid fraction, respectively). The hydrogen peroxide scavenging ability of the Ag-NPs was also examined. Glycoside-mediated-Ag-NP had the highest scavenging activity (28.89%), compared with Ag-NPs biofabricated by alkaloid fraction (21.86%) and lichen aqueous extract (7.21%).

**Figure 5:**
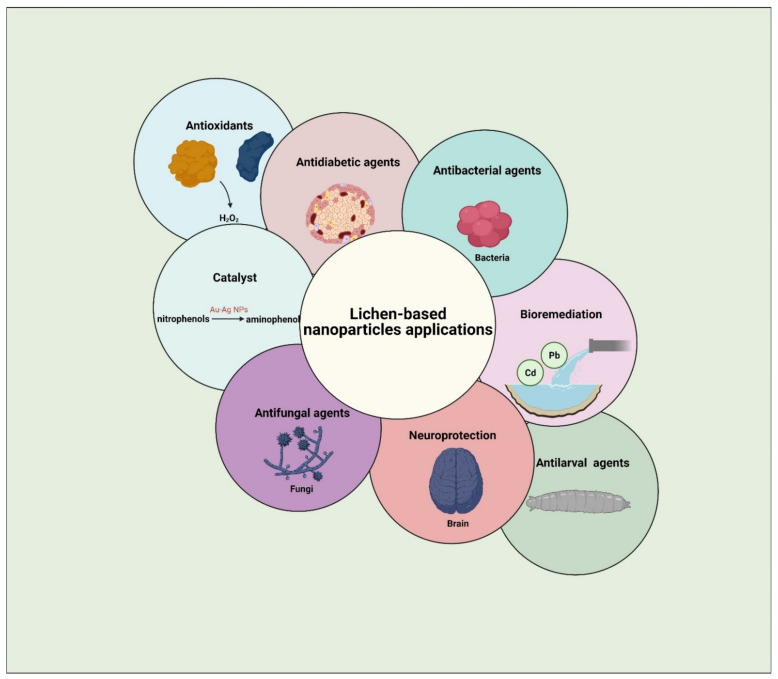
Application of lichen-based nanoparticles.

*Parmelia sulcata* extract (PSE) and PSE-synthesized Au-NPs were investigated for their mosquitocidal activity against *Anopheles stephensi* and *Anopheles aegypti* mosquito larvae, pupae, adults, and egg hatching [160]. Varying concentrations of the lichen extract (75, 150, 225, 300, and 375 ppm) were tested and deemed toxic against larval instars I–IV and pupae of *A. stephensi* and *Anopheles aegypti*. The registered lethal concentration_50_ (LC_50_) values of instars of *A. stephensi* were: 172.16 ppm (I), 201.39 ppm (II), 219.04 ppm (III), 243.89 ppm (IV), and 288.03 ppm (pupae), and the ones for *Anopheles aegypti* were 281.71 ppm (I), 244.46 ppm (II), 283.90 ppm (III), 330.35 ppm (IV), and 346.99 ppm (pupae). The green-synthesized Au-NPs showed exceptionally high activity against larvae and pupae. At concentrations of 15, 30, 45, 60, and 75 ppm, the Au-NPs presented LC_50_ values of 29.82 ppm (I), 33.83 ppm (II), 37.55 ppm (III), 44.26 ppm (IV), and 50.44 ppm (pupae) for *A. stephensi*, and 34.49 ppm (I), 38.72 ppm (II), 44.72 ppm (III), 51.41 ppm (IV), and 59.00 ppm (pupae) for *A. aegypti*. For the adulticidal experiments, the PSE concentrations were 25, 50, 75, 100, and 125 ppm, while those of the Au-NPs were 10, 20, 30, 40, and 50 ppm. The LC_50_ and LC_90_ values of PSE and Au-NPs for *A. stephensi* were 59.35 and 132.80 ppm, and 22.43 and 49.02 ppm, respectively. For *A. aegypti*, the LC_50_ and LC_90_ values of PSE and Au-NPs were 70.16 and 149.66 ppm, and 24.55 and 52.74 ppm, respectively. Multiple concentrations of PSE and Au-NPs (60, 120, 180, 240, 300, 360, and 420 ppm) were tested for their ovicidal effects, and it was concluded that both *A. stephensi* and *A. aegypti* hinder complete egg hatchability at 360 and 240 ppm, respectively. The Au-NPs impose a high toxicity risk on *A. stephensi* and *A. aegypti*. Importantly, the synthesized Au-NPs worked at a far higher efficacy when compared with the PSE. These experiments proved that PSE-synthesized Au-NPs have a markedly successful mosquitocidal effect against *Anopheles.* The study was concluded to provide evidential support for the use of PSE and Au-NPs as a solution towards mosquito-manifested environments (Figure 5).

## 7. Analysis and Characterization of Nanoparticles

Physicochemical characterization analyses of NP samples are the initial and most significant step following the fabrication process of NPs. These analyses are required to confirm the synthesis of NPs and their unique properties such as increased surface area, stability, crystallinity, charge, dispersion, magnetic, thermal, and optical properties, and morphological features such as shape and size. The techniques utilized include spectroscopic analyses such as UV–visible spectroscopy, FTIR, zeta potential, dynamic light scattering, and nuclear magnetic resonance spectroscopy. These spectroscopic methods estimate the corresponding wavelength ranges of NPs, the functional groups surrounding NPs, and evaluate the charge and hydrodynamic diameter of NPs. X-ray-based analyses such as XRD, X-ray photoelectron spectroscopy (XPS), and energy-dispersive spectroscopy (EDAX or EDS) are performed to reveal the chemical composition and crystal structure and phase of the NPs. Microscopic analyses such as TEM, SEM, high-resolution TEM (HRTEM), and atomic force microscope (AFM) are used to demonstrate the morphological features of NPs [153].

## 8. Nanoparticle-Based Green Synthesis-Regulated Parameters: Clues to Enhance Their Activity

**Figure 6:**
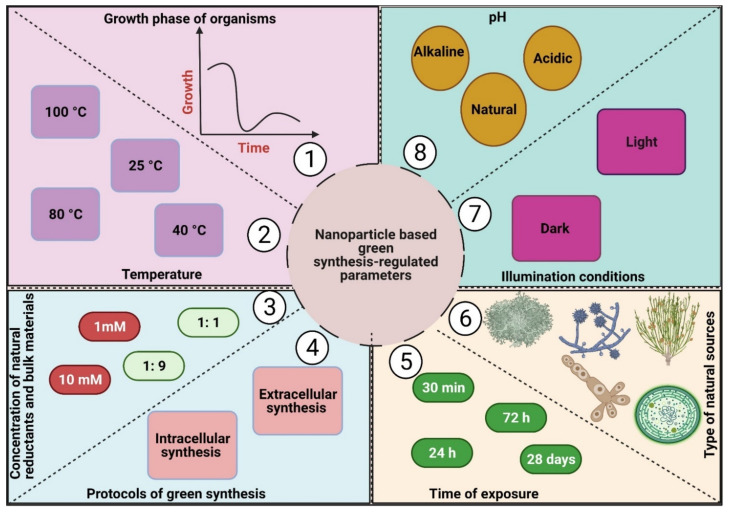
Nanoparticle-based, green synthesis-regulated parameters including (**1**) growth phase of organisms, (**2**) temperature, (**3**) concentrations of reductants and bulk materials, (**4**) protocols of green synthesis, (**5**) time of exposure, (**6**) type of natural sources, (**7**) illumination conditions and (**8**) pH.

Different parameters should be used to optimize synthesis and obtain high efficiency, stabilized, and applicable NPs. These parameters include the type of synthesis method, temperature, pH, time of exposure, concentration of reductants and stabilizing agents, concentration of bulk materials, type of natural sources, illumination, and microorganism growth phase (Figure 6).

### 8.1. Temperature

Temperature is an important factor in controlling the nature of NPs. Generally, biosynthesis processes using natural sources require temperatures ranging from room temperature to 100 °C [172]. Liu et al. synthesized Ag-NPs using *Cinnamomum Camphor* leaf extract at different temperatures including 70, 75, 80, 85, and 90 °C [173]. The temperature had impressively different effects on the nucleation kinetics constant k1 and growth kinetics constant k2 resulting in the generation of NPs with different sizes.

### 8.2. pH

pH is another significant parameter mitigating the green synthesis of NPs. pH influences the size and texture of NPs [174]. Mohammadi et al. synthesized zinc oxide-NPs at different pH (4, 6, 7, 8, and 10) using cherry extract and found that the optimum pH for the fabrication of hexagonal, small NPs was pH 8 (alkaline medium) [175].

### 8.3. Time of Exposure

Wei et al. studied the effect of reaction time (0, 1, 2, 3, and 4 h) on the yield of Ag-NPs using berry extract of sea buckthorn [63]. As the reaction time increased, the absorption intensity of NPs increased steadily, reaching a maximum at 4 h, which was indicative of a high concentration of Ag-NPs. The UV spectra also showed a slight blue shift from 415 to 413 nm with the increase in time of exposure, indicating the formation of smaller-sized NPs.

### 8.4. Concentration of Natural Reductants, Stabilizing Agents, and Bulk Materials

The concentration of biological entities and the salts used for the synthesis of NPs influence the size and shape of the NPs. Hamouda et al. revealed that a surge in the amount of *Oscillatoria limnetica* extract during synthesis of Ag-NPs shifted the UV-spectra peak of the Ag-NPs from 420 to 430 nm, which reflected an increase in the size of the NPs [176]. Similarly, Vellora et al. synthesized copper oxide NPs using different concentrations (1, 2, and 3 mM) of copper chloride (CuCl_2_·H_2_O) and a constant concentration of Gum karya (10 mg/mL) with incubation at 75 °C and 250 rpm for 1 h in an orbital shaker [177]. The increase in the concentration of precursors promoted the generation of NPs of increasing sizes; the nanosizes were 4.8, 5.5, and 7.8 nm, respectively.

### 8.5. Illumination

Light energy is critical for accelerating the reaction rate of NP fabrication. The illumination factor affects the intracellular and extracellular synthesis of NPs using photosynthetic organisms [178]. In contrast, some organisms do not need a light source to synthesize NPs. This phenomenon can be attributed to the fact that some organisms secrete different biomolecules capable of NP fabrication, only some of which need light activation [178]. Light intensity may be another important factor controlling the stability and production of NPs [179]. Recently, Ag-NPs were completely fabricated after 5 min using *Azadirachta indica* leaf extract under sunlight [180].

### 8.6. Protocol of Green Synthesis Method

The type of method(s) used for synthesizing NPs is a crucial parameter controlling the physicochemical and biological properties of NPs. Molnár et al. studied three different methods—extracellular fraction, autolysate of the fungal cells, and intracellular fractions—for synthesizing Au-NPs using 29 thermophilic filamentous fungi [65]. NPs were formed using all three methods; however, the extracellular methods were the most acceptable, yielding NPs with a smaller size and low polydispersity. The authors also recommended washing the fungal mycelia several times before extracellular biofabrication to avoid the influence of residual growth media components on the NP synthesis process.

### 8.7. Type of Natural Sources

The nature of organisms used in NP fabrication processes significantly influences the nature of the resulting NPs. Biomolecules such as pigments, proteins, polysaccharides, etc. vary between different organisms and in strains of the same species, and this leads to variations in an organism’s potentiality to produce NPs [30]. Recently, Ag-NPs were produced using three different strains (*Nostoc* sp. Bahar M [58], *Nostoc* HKAR-2,98 [181], and *Nostoc muscorum* NCCU-442,56 [60]). These strains produced Ag-NPs with a range of different sizes including 8.5–26.44 mm, 51–100 mm, and 42 nm, respectively.

### 8.8. Growth Phase of Organisms Used for NP Fabrication

The effect of the growth phase on the fabrication process of NPs was studied by Sweeney et al. [182]. Cadmium sulfide nanocrystal production varies dramatically depending on the growth phase of *E. coli*. The formation of NPs increased approximately 20-fold in the stationary phase of *E. coli*, compared with that grown in the late logarithmic phase.

## 9. The Mechanism of Biological Synthesis of NPs

Different speculations about the mechanism of NP synthesis using living organisms were reported, but until now, the exact mechanism remained unclear. However, each organism was found to have its own synthesis mechanism against different metals [4,183]. One hypothesis is the ability of living organisms to synthesize NPs occurs via two general steps—(i) metal ions are trapped on the surface of an organism and/or inside their cells and (ii) these ions are reduced to NPs aided by biomolecules such as enzymes, proteins, pigments, or polysaccharides, or by the union effect of different molecules [4,67,183,184]. These biological molecules are responsible for the electron shuttle reduction and stabilization of NPs. Sneha et al. reported that metal ions, particularly Au or Ag ions, are captured on the fungal cell surface through the electrostatic force between the metal ions and cell wall, which carries a negative charge from the enzyme carboxylate groups. The enzymes then fabricate the metals into Au or Ag nuclei, which sequentially grow by reduction and accumulation [185]. Kalishwaralal et al. reported that nitrate reductase produced by *Bacillus licheniformis* facilitates the bioreduction of Ag-NPs. The nitrate ions of silver nitrate were found to activate the nitrate reductase enzyme, resulting in reducing silver ions to metallic silver via an electron shuttle enzymatic metal reduction process [186]. During the biosynthesis of MNPs, NADH and NADH-dependent nitrate reductase enzymes (especially nitrate reductase) are essential factors [187]. These enzymes are known to be secreted from *B. licheniformis* and may be linked to the biofabrication of Ag^+^ to Ag^0^ and subsequent synthesis of Ag-NPs. Divya et al. reported that the existence of NADH and NADH-based reductases in the supernatant of *Alcaligenes faecalis* was responsible for the reduction of silver nitrate into Ag-NPs [188]. Hamedi et al. studied the synthesis of Ag-NPs using *Fusarium oxysporum66* cell-free culture filtrates [189]. They reported that a surge in C:N ratio resulted in the enhancement of the nitrate reductase activity, causing an increase in the Ag-NPs fabrication process rate. Furthermore, they obtained small Ag-NPs with a narrow size distribution.

Exopolysaccharides (EPSs) are predominantly composed of carbohydrates (such as D-glucose and D-mannose) and noncarbohydrate components (such as carboxyl, phosphate, and sulfate), which characterize them with anionic properties. These organic molecules enhance the lipophilicity of EPSs and directly influence their interaction with polysaccharides and cations. It was found that EPSs chelate metal ions. Then, sugar molecules of EPSs reduce metal ions into NPs to be capping with different functional groups of EPSs [190,191,192].

Kang et al. synthesized Ag-NPs using EPSs in an *E. coli* biofilm. They reported that EPSs aldehyde and hemiacetal groups of rhamnose sugars were responsible for the reduction and stabilization of NPs [193].

Moreover, the synthesis of heavy MNPs can be due to the genetic and proteomic responses of a metallophilic microorganism to toxic environments [194]. Heavy metal ions such as mercury, cadmium, zinc, and copper ions are a hazard to microbial survival and consequently, microorganisms have developed genetic and proteomic responses to tackle these threats [195]. Microorganisms contain many gene clusters of metal resistance that enable cell detoxification via different mechanisms, such as complexation, efflux, or reductive precipitation [196]. Recently, a mechanism for the synthesis of magnetites using *Shewanella oneidensis* was suggested and comprised both passive and active processes [197]. Initially, Fe^2+^ is actively produced when bacteria use ferrihydrite as a terminal electron acceptor, accompanied by elevation in the pH round the cells due to the bacterial metabolism of amino acids. The passive mechanism then uses the localized concentration of Fe^2+^ and Fe^3+^ at the net negatively charged cell wall, cell structures, and/or cell debris, which enhances a local rise of supersaturation of the system with respect to magnetite, resulting in precipitation of the magnetite phase.

**Figure 7:**
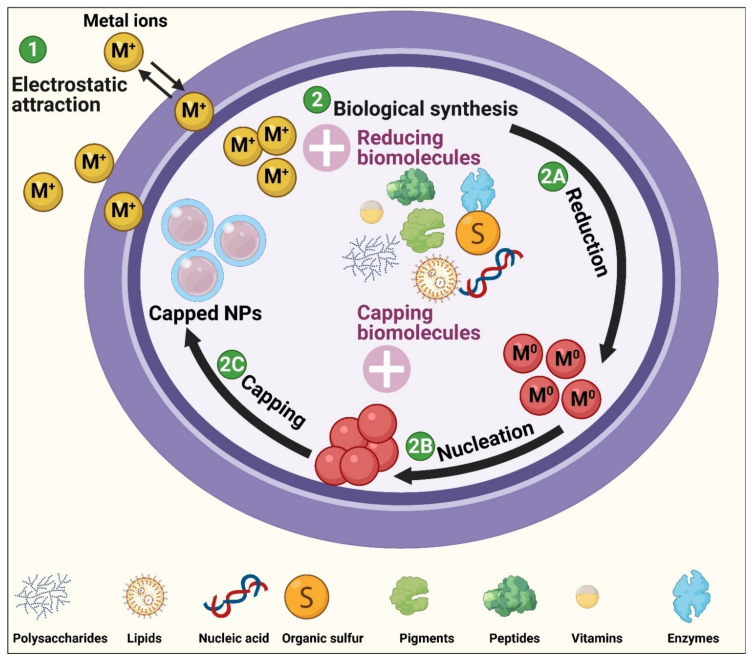
The potential mechanism of biological synthesis of NPs.

Another hypothesis discussed the role of c-type cytochromes redox proteins for electron transfer during the reduction of metals. Ng et al. synthesized Ag-NPs using a mutant strain of *Shewanella oneidensis* missing cytochrome genes (MtrC and OmcA) and a wild-type strain of *S. oneidensis*. They found that c-type cytochromes aid in electrons transfers to metal ions outside the cells. Similarly, Liu et al. reported that c-type cytochrome protein complexes (ombB, omaB, and omcB) in the outer membrane of metal-reducing bacterium Geobacter sulfurreducens PCA was responsible for the extracellular reduction of Fe (III)-citrate and ferrihydrite (Figure 7).

## 10. Toxicity of Nanoparticles

Although nanotechnology is rapidly growing with a wide range of applications in different areas such as industry, agriculture, medicine, biotechnology, etc., there remain many barriers with this technology such as the toxicological effects of NPs on ecology and living organisms. Many in vitro and in vivo investigations revealed that metallic and non-metallic NPs have serious side effects on human health and the environment. Senut et al. reported that mercaptosuccinic acid-capped Au-NPs (1.5 nm) at a concentration of 0.1 μg/mL enhanced cell death of human embryonic stem cells (ESCs). However, other Au-NPs (4 and 14 nm) at the same concentration showed almost no toxic effect on ESCs [198]. Chen et al. studied the relation between the toxicity of Ag-NPs and their size against fresh red blood cells. The scholars used three different characteristic sizes (15, 50, and 100 nm) of Ag-NPs [199]. They reported that smaller sizes of Ag-NPs enhancing the hemolysis and membrane damage of blood cells, compared with that of other sizes. Wan et al. investigated the genotoxicity of the chemically synthesized cobalt NPs in vivo by utilizing guanine phosphoribosyltransferase delta transgenic mice [200]. The authors reported that cobalt NPs induced oxidative stress, lung inflammation and injury, DNA damage, and mutation.

The toxic effects of TiO_2_ NPs against human cells, vertebrates, and invertebrate animals might be attributed to their potency to form free radicals with water in the presence of sunlight. TiO_2_ NPs caused DNA damage with or without light and induced the cell death pathway in hamster fibroblasts with stretched micronuclei [201,202]. Although biological synthesis processes have become a simple, eco-friendly, low-cost alternative to traditional methods of NPs fabrication, there are few studies in the literature discussing their toxicity on humans and the environment. Some investigations reported the biocompatibility of green NPs, compared with that synthesized by chemical and physical methods. This could be assumed to the synergetic effect between synthesized NPs and their biological coats [203]. Devasena et al. reported that Mg-NPs synthesized by *Cladonia rangiferina* extract exhibited a better antimicrobial activity and lower toxicity [161]. Khorrami et al. reported that Ag-NPs synthesized by walnut green husk (as reducing and capping agents) showed high selectivity toward breast cancer cells (MCF-7) than normal cells (L-929) [203]. However, the chemically synthesized Ag-NPs showed high toxicity against L-929 cells, compared with biologically synthesized Ag-NPs. Ag-NPs formed by biological synthesis using *Lycium chinense* fruit extract showing low cytotoxicity against normal murine macrophage RAW264.7 cells [204]. On the other hand, Krishnaraj et al. exhibited that Ag-NPs synthesized by *Malva crispa* plant caused morphological alterations in adult zebrafish gills and reduced the biological connection and the homogenous distribution of their liver parenchyma cells [205]. To fill the gap in knowledge regarding this association, many additional in vitro and in vivo investigations are required to test the toxicity of green NPs against normal cells, explore the biocompatibility of biogenic NPs, and determine the precise lethal mechanism of NPs against living cells to increase the potentiality of using these NPs as FDA-approved drugs.

## 11. Future Prospects and Conclusions

The current review discussed the biological synthesis methods in depth with emphasis on the lichen-mediated synthesis of NPs. The biological synthesis of NPs has recently become an increasingly active area of research. Through exhibiting several advantageous qualities and numerous potential applications, biological synthesis methods of NPs have proven to be superior to the traditional chemical and physical methods. These qualities include being cost effective, eco-friendly, and vastly applicable in biomedical fields due to biocompatibility. Lichen species are seldom considered as biomachinery for the biofabrication of NPs. In this review, we have highlighted the potential of these organisms as natural biofactories for NP formation. The symbiosis between fungi and cyanobacteria or algae or sometimes plants makes lichen a promising alternative biomachinery for NPs fabrication. Due to the variation in their biomolecule contents and structures, which are responsible for reducing metal ions into NPs. Devasena et al. reported that lichen-mediated synthesis NPs are distinguished from other alternative biological methods by being lesser toxic and needing low-processing conditions [161]. Lichen species have a reducible activity to fabricate different types of NPs, including gold (Au)-NPs, silver (Ag)-NPs, metal oxide-NPs such as iron oxide- and zinc oxide-NPs, and other nanomaterials such as bimetallic alloys (Au-Ag NPs) and nanocomposites such as ZnO@TiO_2_@SiO_2_ and Fe_3_O_4_@SiO_2_. These biogenic NPs have significant antimicrobial activities against both Gram-positive and Gram-negative bacteria, and fungi, and they also display mosquitocidal activity. Additionally, these NPs act as potential catalytic materials, bioremediatory agents for heavy metals, antidiabetics, antioxidants, and neuroprotection agents against neurotoxin.

Extending the utilization of lichen-mediated green synthesis methods and exploring the optimum conditions of these processes to fabricate applicable, bioactive, scalable, and biocompatible nanoproducts may lead to the development of novel green NPs with unique physicochemical and biological features that can be applied in different sectors, including agriculture, industry, medicine, biotechnology, and pharmaceutics. Moreover, there remain many barriers against the biological synthesis process, including toxicity and agglomeration, polydispersity, stability, and the nonuniform size of NPs. These issues can be solved by increasing the optimization studies for green synthesis of NPs to obtain the desirable NPs. Additionally, exploring the synthesis mechanism of NPs using natural sources will facilitate the development and launch of nanodrugs in different fields.

## Figures and Tables

**Table 1:** Lichen-based synthesis of nanoparticles (NPs).

Strains	Type of NPs	Size (nm)	Shape	Illumination	Time of Exposure	pH	Temperature (°C)	Mode of Synthesis	Application	Reference
***Usnea longissima***	Ag-NPs	9.40–11.23	Spherical	Dark	72 h	7	RT	-	Antibacterial agent	[136]
***Parmotrema praesorediosum***	Ag-NPs	19	Cubic structure	NM	24 h	NM	RT	-	Antibacterial agent	[152]
***Cetraria islandica (L) Ach***	Ag-NPs	5-29	Spherical	NM	19.09, 60 120, 180 and 220.91 min	NM	16.48, 25, 37.5, 50, 58.52	-	NA	[139]
***Parmotrema praesorediosum***	Ag-NPs	42	Spherical	NM	72 h	Alkaline	RT	-	NA	[151]
***Ramalina dumeticola***	Ag-NPs	20	Spherical	NM	72 h	Alkaline	RT	-	NA	[151]
***Ramalina dumeticola***	Ag-NPs	13	Spherical	NM	24 h	NM	RT	-	NA	[154]
***Cetraria islandica (L.) Ach***	Ag-NPs	6	Spherical	NM	30 min	NM	80	-	Catalytic activity	[144]
	Au-NPs	19	Spherical	NM	30 min	NM	80	-		
	Ag-Au NPs	6 and 21	Polygonal and Spherical	NM	30 min	NM	80	-		
***Parmelia perlata***	Ag-NPs	NA	Spherical	NM	30 min	NM	60	-	Antimicrobial, antioxidant and antidiabetic agents	[150]
***Ramalina sinensis***	Fe_3_O_4_ NPs	20-40	Uniform Spherical	NM	1 h	NM	70	-	Removing heavy metals such as Pb and Cd	[163]
***Lecanora muralis***	ZnO@TiO2@SiO2 nanocomposites	55–90	Spherical	NM	5 h	NM	80	-	Antimicrobial agent	[164]
	Fe3O4@SiO2 nanocomposites	55–85	Spherical	NM	5 h	NM	80	-		
***Ramalina sinensis***	Iron oxide nanoparticles	31.74-53.91	Uniform spherical	NM	1 h	7	70	-	Antibacterial agent	[165]
***Protoparmeliopsis muralis***	Ag-NPs	33.49 ± 22.91	Spherical	NM	24 h	8	RT	-	Antibacterial, antibiofilm, antiquorum sensing, antimotility, and antioxidant activities	[162]
	Cu-NPs	253.97 ± 57.2	Triangular	NM	24 h	8	RT	-		
	Fe3O4 NPs	307 ± 154	Spherical	NM	24 h	8	RT	-		
	TiO_2_ NPs	133.32 ± 35.33	Polyhedral	NM	24 h	8	RT	-		
	ZnO NPs	178.06 ± 49.97	Cubic	NM	24 h	8	RT	-		
***Parmeliopsis ambigua***	Ag-NPs	150–250	NM	Light	24 h	NM	RT	+, -	Antibacterial and antioxidant agents	[153]
***Punctelia subrudecta***	Ag-NPs	150–250	NM	Light	24 h	NM	RT	+, -		
***Evernia mesomorpha***	Ag-NPs	150–250	NM	Light	24 h	NM	RT	+, -		
***Xanthoparmelia plitti***	Ag-NPs	150–250	NM	Light	24 h	NM	RT	+, -		
***Cladonia rangiferina***	Mg-NPs	23	NM	NM	24 h	NM	NM	NM	NA	[161]
***Parmotrema tinctorum***	Ag-NPs	15 ± 5.1	Spherical	Dark	24 h	NA	RT	-	Antibacterial agent	[149]
***Acroscyphus sphaerophoroides***	Ag-NPs	5–35	Twinned quasi-spherical and prismatic shapes	NM	12 h	NM	RT	-	Antioxidant agent	[159]
***Sticta nylanderiana***	Ag-NPs	20–50	Multiply twinned	NM	12 h	NM	RT	-		
***Parmotrema clavuliferum***	Ag-NPs	106	Spherical	Dark	48 h	NM	80 °C	-	Antibacterial agent	[158]
***Parmotrema perlatum***	Ag-NPs	NM	NM	NM	NM	NM	NM	NM	Antibacterial agent	[166]
***Xanthoria parietina***	Ag-NPs	1–40	Spherical	Dark	72 h	NM	40 °C	-	Anticancer and antibacterial agents	[37]
***Flavopunctelia flaventior***	Ag-NPs	1–40	Spherical	Dark	72 h	NM	40 °C	-		
***Parmelia perlata***	Ag-NPs	NM	NM	NM	NM	NM	NM	NM	Antibacterial agent	[167]
***Umbilicaria Americana***	Ag-NPs	NM	NM	NM	NM	NM	NM	NM	NM	[168]
***Cladonia rangiferina***	Ag-NPs	20	Spherical and rods	NM	72 h	Alkaline	RT	-	Antibacterial agent	[145]
***Usnea articulata***	Ag-NPs	10–50	Spherical	NM	72 h	Alkaline	27 °C	-	Antibacterial agent	[143]
***Ramalina sinensis***	Ag-NPs	50–80	Spherical	NM	72 h	Alkaline	27 °C	-		
***Parmelia sulcate***	Au-NP	54	Spherical	NM	20 min	NM	60 °C	-	Antioxidant and mosquitocidal agents	[160]
***Ramalina fraxinea***	ZnO-NPs	21	Spherical	NM	Up to 2 h	NM	60 °C	-	Neuroprotection activity	[169]
***Aspicilia lichens***	Nanohyaluronic acid	29–89	Spherical	NM	48 h	Alkaline then neutralize by acid	50 °C	-	Antidiabetic agent	[146]
***Xanthoria elegans***	Ag-NPs	Bimodal	5-100	NM	2 h	NM	NM	-	Antibacterial agent	[155]
***Usnea antarctica***		Bimodal	5-100	NM	6 h	NM	NM	-	Antibacterial agent	
***Leptogium*** ***puberulum***		Bimodal	5-100	NM	6 h	NM	NM	-	Antibacterial agent	
***Cetraria islandica***		Bimodal	5-100	NM	2 h	NM	NM	-	Antibacterial agent	
***Pseudevernia*** ***furfuracea***	Ag-NPs	Bimodal	˂10-100	NM	2 h	NM	NM	-	Antibacterial and antioxidant agents	[156]
***Lobaria pulmonaria***		Bimodal	˂10-100	NM	2 h	NM	NM	-	Antibacterial and antioxidant agents	
***Heterodermia boryi***	Ag-NPs	Cubic	27.91–37.21	NM	NM	NM	NM	-	Antibacterial agent	[157]
***Parmotrema*** ***stuppeum***		Cubic	27.69–36.00	NM	NM	NM	NM	-	Antibacterial agent	

**Abbreviation:** (-), extracellular synthesis; (+), intracellular synthesis; NM, not mentioned; NA, no applications; RT, room temperature.

## Data Availability

The data supporting this article are shown in Figure 1, Figure 2, Figure 3, Figure 4, Figure 5, Figure 6 and Figure 7 and one table. The data sets analyzed in the present study are available from the corresponding author upon reasonable request.
